# Distinct regulatory networks control toxin gene expression in elapid and viperid snakes

**DOI:** 10.1186/s12864-024-10090-y

**Published:** 2024-02-16

**Authors:** Cassandra M. Modahl, Summer Xia Han, Jory van Thiel, Candida Vaz, Nathan L. Dunstan, Seth Frietze, Timothy N. W. Jackson, Stephen P. Mackessy, R. Manjunatha Kini

**Affiliations:** 1 Department of Biological Sciences, Faculty of Science, National University of Singapore, Singapore, Singapore; 2 Centre for Snakebite Research and Interventions, Liverpool School of Tropical Medicine, Liverpool, U.K.; 3 Fulcrum Therapeutics, Cambridge, MA U.S.A.; 4 Institute of Biology Leiden, Leiden University, Leiden, The Netherlands; 5 Human Development, Institute for Clinical Sciences (SICS), Agency for Science, Technology and Research (A*STAR), Singapore, Singapore; 6 Venom Supplies, Tanunda, South Australia Australia; 7 Department of Biomedical and Health Sciences, University of Vermont, Burlington, VT U.S.A.; 8 Australian Venom Research Unit, Department of Biochemistry and Pharmacology, University of Melbourne, Melbourne, Australia; 9 Department of Biological Sciences, University of Northern Colorado, Greeley, CO U.S.A.; 10 Department of Pharmacology, Yong Loo Lin School of Medicine, National University of Singapore, Singapore, Singapore; 11 Singapore Eye Research Institute, Singapore, Singapore; 12 Department of Biochemistry and Molecular Biology, Virginia Commonwealth University, Richmond, VA U.S.A.

**Keywords:** Front-fanged snakes, Venom delivery systems, Epigenetics, Toxin promoters

## Abstract

**Background:**

Venom systems are ideal models to study genetic regulatory mechanisms that underpin evolutionary novelty. Snake venom glands are thought to share a common origin, but there are major distinctions between venom toxins from the medically significant snake families Elapidae and Viperidae, and toxin gene regulatory investigations in elapid snakes have been limited. Here, we used high-throughput RNA-sequencing to profile gene expression and microRNAs between active (milked) and resting (unmilked) venom glands in an elapid (Eastern Brown Snake, *Pseudonaja textilis*), in addition to comparative genomics, to identify *cis-* and *trans-*acting regulation of venom production in an elapid in comparison to viperids (*Crotalus viridis* and *C. tigris*).

**Results:**

Although there is conservation in high-level mechanistic pathways regulating venom production (unfolded protein response, Notch signaling and cholesterol homeostasis), there are differences in the regulation of histone methylation enzymes, transcription factors, and microRNAs in venom glands from these two snake families. Histone methyltransferases and transcription factor (TF) specificity protein 1 (Sp1) were highly upregulated in the milked elapid venom gland in comparison to the viperids, whereas nuclear factor I (NFI) TFs were upregulated after viperid venom milking. Sp1 and NFI *cis-*regulatory elements were common to toxin gene promoter regions, but many unique elements were also present between elapid and viperid toxins. The presence of Sp1 binding sites across multiple elapid toxin gene promoter regions that have been experimentally determined to regulate expression, in addition to upregulation of Sp1 after venom milking, suggests this transcription factor is involved in elapid toxin expression. microRNA profiles were distinctive between milked and unmilked venom glands for both snake families, and microRNAs were predicted to target a diversity of toxin transcripts in the elapid *P. textilis* venom gland, but only snake venom metalloproteinase transcripts in the viperid *C. viridis* venom gland. These results suggest differences in toxin gene posttranscriptional regulation between the elapid *P. textilis* and viperid *C. viridis.*

**Conclusions:**

Our comparative transcriptomic and genomic analyses between toxin genes and isoforms in elapid and viperid snakes suggests independent toxin regulation between these two snake families, demonstrating multiple different regulatory mechanisms underpin a venomous phenotype.

**Supplementary Information:**

The online version contains supplementary material available at 10.1186/s12864-024-10090-y.

## Background

Snake venom glands are highly specialized vertebrate secretory tissues, exhibiting elevated, tissue-specific expression of 50–100 or more toxins. Based on structural similarity to cognate proteins involved in physiological processes, snake toxins are hypothesized to have primarily evolved by gene duplication of nontoxic genes [1–9] and subsequent neofunctionalization through accelerated evolution of their exons [10–13]. Therefore, snake venom glands provide a unique opportunity to study epigenetic and genetic mechanisms that regulate tissue-specific expression and gene neofunctionalization in multi-locus gene families. Furthermore, as venom glands are a rich source of diverse bioactive proteins applicable to bioprospecting and antivenom development, understanding how toxins are produced would also benefit large-scale production in vitro [14].

Snakebite annually leads to the death or disability of over 500,000 victims [15] and medically significant species are predominantly from the families Elapidae and Viperidae. Venoms from these two families cause debilitating effects and appear to share a common evolutionary origin, which is supported by anatomical and developmental evidence [16], and phylogenetic analysis of toxins suggests venom evolved once, at the base of the advanced snake radiation [17]. Although elapids and viperids both have tubular fangs positioned anterior on the maxilla, elapid and viperid delivery systems and venom components are distinct in several ways. Elapids have a less mobile maxillary bone, and relatively smaller fangs and venom gland lumen, while viperids have a highly kinetic maxillary, long fangs and a wide lumen [18, 19]. Different external adductor muscles are also used to compress the venom gland in elapids and viperids [20], as well as likely convergent evolution of accessory glands [21]. After venom is expelled, venom gland cells do not exhibit any marked size differences in elapids [22, 23]; while in viperids, secretory epithelial cells dramatically increase in size from cuboidal to columnar with proliferation of the rough endoplasmic reticulum (ER) [24, 25]. Further, elapid venoms are largely dominated by non-enzymatic three-finger toxins (3FTxs), and enzymatic group I phospholipases (PLA_2_s) that share homology with secretory PLA_2_ from the pancreas [26, 27], while viperid venoms have primarily enzymatic toxins, including snake venom metalloproteinases (SVMPs), serine proteinases (SVSPs), and group II PLA_2_s with homology to non-pancreatic inflammatory PLA_2_s [28, 29]. Chromosomal locations of dominant toxins also vary between these two snake families, with most located on macrochromosomes in elapids [9, 30] and microchromosomes in viperid snakes [31–33].

In both elapids and viperids, toxin gene transcription and translation are upregulated when the venom gland is emptied by venom extraction or “milking” [23, 34–36]. Studies investigating mechanisms regulating the expression of elapid toxins have focused on a small number of genes. These studies have identified *cis*-regulatory elements (CRE) that regulate the expression of genes encoding 3FTx and group I PLA_2_ expression in the Javan Spitting Cobra (*Naja sputatrix*) [37–39] and the pseutarin C catalytic subunit in the Eastern Brown Snake (*Pseudonaja textilis*) [40]. Additionally, silencer AG-rich motifs have also been identified in the first intron of the pseutarin C catalytic subunit gene in *P. textilis* [41]. For the viperid Jararaca (*Bothrops jararaca*), α_1_− and β−adrenoceptor signaling activates transcription factors (TFs) NF-κB and AP-1 to initiate toxin synthesis [42, 43]. Habu (*Protobothrops flavoviridis*) tissue-specific TF ESE-3 activates group II PLA_2_ gene promoters [44]. Prairie and Tiger rattlesnakes (*Crotalus viridis* and *C. tigris*, respectively) have GRHL- and NFI-family TF binding sites enriched within SVMP, SVSP, and group II PLA_2_ gene promoters [31, 32, 45], in addition to co-opted TFs from the Unfolded Protein Response (UPR) pathway, that are potentially all involved in toxin gene regulation [45]. Current evidence suggests that for the viperids *C. viridis* and *C. tigris*, toxin gene expression is regulated by similar mechanisms as have been observed for non-toxin genes: chromatin organization, TFs, and gene methylation levels [31, 32]. Here, we compare toxin gene expression in active (milked) and resting (unmilked) venom glands of *P. textilis*, and between this elapid and viperids, providing the first direct comparison of gene regulatory networks between these two snake families.

*Pseudonaja textilis* is the second most lethal terrestrial venomous snake in the world, based upon murine models of toxicity [46, 47], and is responsible for the majority of snakebite deaths in eastern Australia [48, 49]. Venom gland transcriptomics and venom proteomics have been used to profile *P. textilis* venom composition [50–52], and studies have documented geographic, seasonal, ontogenetic, and individual venom variation [53–59]. Using high-throughput transcriptomics, we evaluated *P. textilis* mRNAs and microRNAs (miRNAs) in a Milked Venom Gland (MVG) and Unmilked Venom Gland (UVG) from an individual snake, eliminating contributing factors involved in venom variation, to identify mechanisms regulating toxin expression. We identified higher-level venom synthesis activation pathways common to both *P. textilis* and viperid venom glands, but differences in *cis-* and *trans-*acting regulation of toxin expression. Further, posttranscriptional miRNA regulation was not conserved between venom glands from the two snake families. Therefore, distinct gene regulatory networks produce elapid and viperid venom phenotypes, and thus, our results support the conclusions of an independent origin of toxin genes between these two snake families [60].

## Results

### Toxins exhibit high and variable isoform expression in the *Pseudonaja textilis* venom gland

From a *P. textilis* individual, the left venom gland was milked to stimulate venom production while the right venom gland remained unmilked as an ‘unstimulated’ control. Four days later (96 h post venom milking, hpvm), both the MVG and UVG were collected, RNA and miRNA isolated and sequenced, and the genome (EBS10Xv2-PRI) used as a reference to profile gene expression between glands. Toxin genes in the *P. textilis* genome are not well annotated and given that venom variation is also present between individuals, we therefore performed *de novo* transcriptome assemblies for each venom gland to compare toxin transcript diversity and expression (Supplemental Table 1). Fold-changes in transcript expression between the MVG versus UVG were determined with GFOLD [61] (Supplemental Table 2). Of a total of 34,668 transcripts, 7,062 transcripts were upregulated and 8,038 transcripts were downregulated in the MVG.

For both the MVG and UVG, toxins were among the most highly expressed transcripts and the same toxin superfamilies made up similar proportions of overall toxin expression (Supplemental Fig. 1A-B). From a combined *de novo* assembly of each gland (Supplemental Table 1), 28,569 transcripts were expressed (> 1 Transcript Per Million, TPM), and 52 full-length toxins from 18 toxin superfamilies were identified. Toxin superfamilies that were expressed, in order of total abundance in the MVG, include fourteen 3FTxs (78% of all toxin transcripts), three group I PLA_2_s (13%), four Kunitz-type serine proteinase inhibitors (KUNs, 3%), 13 snake venom C-type lectins (Snaclecs, 2%) and the prothrombin activator pseutarin C (venom coagulation factors V and X, 2%) (Supplemental Fig. 1A). Low abundance toxins, making up 1% or less of reads, were two natriuretic peptides (NPs,), one cysteine-rich secretory protein (CRISP), three cystatins (CYSs), two SVMPs, one hyaluronidase (HYAL), one nerve growth factor (NGF), one waprin (WAP), one 5’-nucleotidase (5’NUC), one cobra venom factor (CVF), one L-amino acid oxidase (LAAO), one acetylcholinesterase (AChE) and one vespryn (VES). All transcript isoforms in a superfamily were not expressed equally; for the two superfamilies with the highest expression levels, the fourteen 3FTx isoforms ranged from 236,773 TPM to 10 TPM, and the three PLA_2_ isoforms ranged from 100,141 TPM to 1,846 TPM.

This *de novo* assembled venom gland transcriptome is currently the most comprehensive to date for *P. textilis*, having detected all previous reported toxin transcripts along with full-length transcripts for low abundance CYSs, HYAL, NGF, WAP, 5’NUC, CVF, LAAO, AChE, and VES toxins (Supplemental Table 3). Three 3FTxs (3FTx_6, 7, and 8), one CRISP (CRISP_1), one KUN (KUN_1), and one PLA_2_ (PLA2_1) were identical to previously identified sequences (Supplemental Fig. 2A-D). PLA_2_s and short-chain 3FTxs exhibited the greatest sequence variation (as low as 43% and 48% amino acid sequence identity, respectively) between isoforms. There was an absence of transcripts sequences for textilotoxin, a pentameric PLA_2_ complex unique to *P. textilis* venom [62]. Pseutarin C, a toxin composed of two subunits homologous to the mammalian coagulation factor Xa-Va complex [4], were highly conserved, 99% and 95% identical to the published sequences for *P. textilis* venom coagulation factors V and X, respectively [63, 64] (Supplemental Fig. 2E-F), and > 84% identical to venom coagulation factor homologs in *Oxyuranus microlepidotus* and *O. scutellatus* venom.

### Upregulation of toxin gene expression after venom milking is lower in *Pseudonaja textilis* compared to viperids

To compare changes in toxin gene expression in the MVG of an elapid to that of vipers, we retrieved available RNA-seq libraries from the National Center for Biotechnology Information (NCBI) for MVGs and UVGs of *C. viridis* at 0 and 72 hpvm [32], and *C. tigris* at 0, 72 and 96 hpvm [31]. To have a comparable time course of 0 to 96 hpvm for all species, we milked venom from a *C. viridis* individual of the same northeastern Colorado locality as snakes used by Schield et al. (2019), collected both venom glands at 96 hpvm, and performed RNA-seq on these tissues. Annotated transcriptomes from genome assemblies (UTA_CroVir_3.0 and ASM1654583v1 for *C. viridis* and *C. tigris*, respectively) and myotoxin transcripts from a *de novo* assembled venom gland transcriptome for *C. viridis* (96 hpvm individual) (Supplemental Table 4) were used as references to determine toxin transcript abundances, the myotoxin transcripts were retained because this toxin gene hadn’t yet been annotated in the genome. We reanalyzed fold-changes in expression of transcripts between MVGs and UVGs at each time point with GFOLD [61] (Supplemental Table 7), allowing for comparisons to the *P. textilis* dataset.

**Fig. 1:**
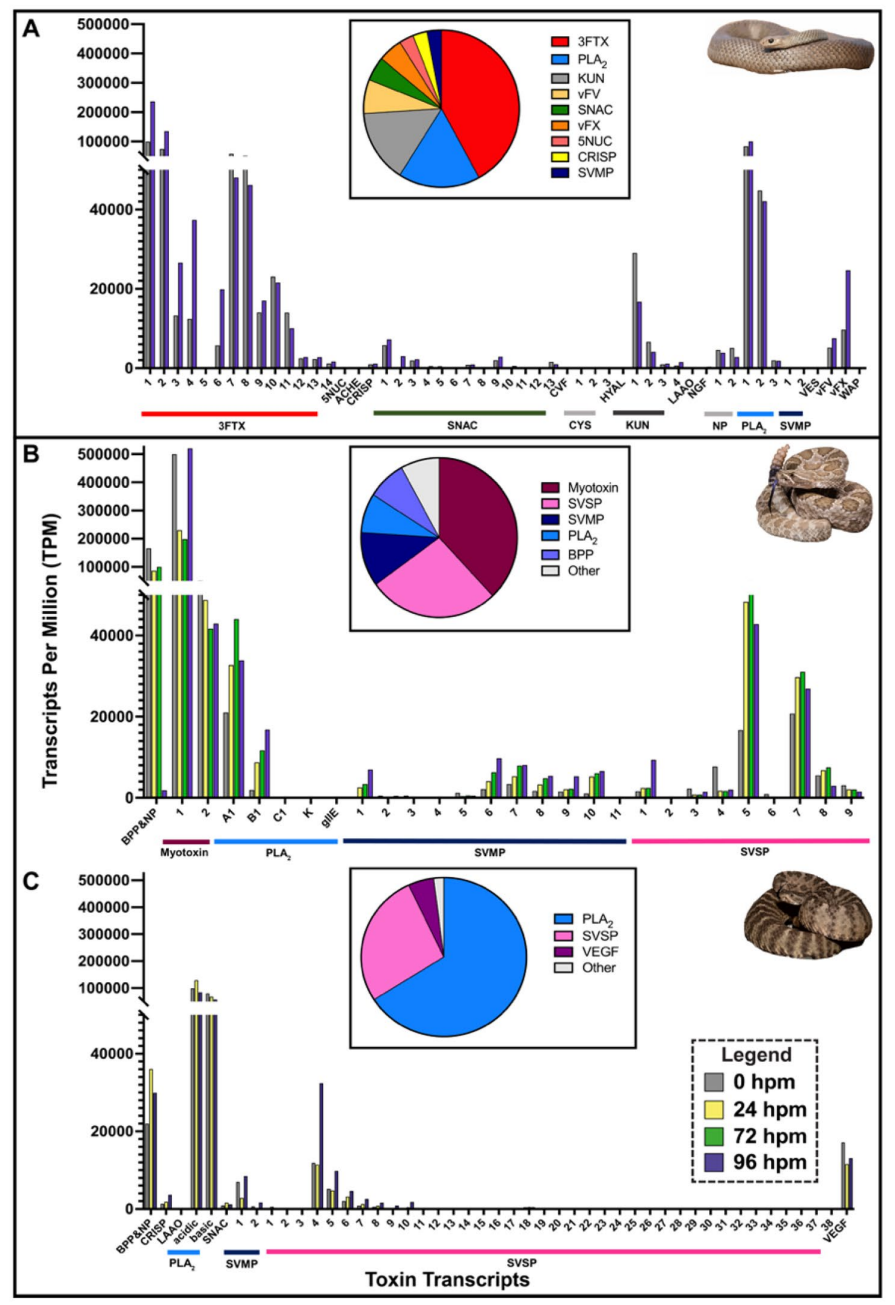
Toxin gene expression in an elapid (*Pseudonaja textilis*) and two viperids (*Crotalus viridis* and *C. tigris*) over a time course of 96 hours post venom gland milking (hpvm) in comparison to venom proteomes. Time points are shown for (**A**) *P. textilis* at 0 and 96 hpvm, (**B**) *C. viridis* at 0, 24, 72, and 96 hpvm, and (**C**) *C. tigris* at 0, 24, and 96 hpvm. Bar colors represent the time after venom milking. Pie charts (insets in respective panels) correspond to the venom proteomes of *P. textilis* [54], *C. viridis* [70] and *C. tigris* [68]. Toxin identifications are as follows: 3FTx = three-finger toxin, 5NUC = 5’-nucleotidase; AChE = acetylcholinesterase; BPP = Bradykinin-potentiating peptide; CRISP = cysteine-rich secretory protein; CVF = cobra venom factor; CYS = cystatin; HYAL = hyaluronidase; KUN = Kunitz-type serine proteinase inhibitor; LAAO = L-amino acid oxidase; NGF = nerve growth factor; NP = natriuretic peptide; PLA2 = phospholipase A_2_; SNAC = snake venom C-type lectin; SVMP = snake venom metalloproteinase; SVSP = snake venom serine protease; VEGF = vascular endothelial growth factor; VES = vespryn; vFV = venom factor V (pseutarin C non-catalytic subunit); vFX = venom factor X (pseutarin C catalytic subunit); WAP = waprin. Photo credits: *P. textilis*, Ákos Lumnitzer; *C. viridis*, Wolfgang Wüster; *C. tigris*, Ben Lowe

Toxin expression in the venom gland of *C. viridis* from northeastern Colorado was predominately myotoxins (75% of total toxin transcripts), SVSPs (11%), PLA_2_s (7%), SVMPs (6%), and minor toxins (< 1%) (Supplemental Table 5). For *C. tigris*, PLA_2_s were the highest expressed (56% of all toxin transcripts), followed by SVSPs (21%), bradykinin-potentiating peptides (BPPs; 12%), vascular endothelial growth factor (VEGF; 5%), SVMPs (4%), and all other minor toxins (1% or less) (Supplemental Table 6). A myotoxin from *C. viridis* had the highest expression level (520,544 TPM at 96 hpvm) of any toxin from either the two rattlesnake venom glands or from the elapid *P. textilis* (Fig. 1). For all species, highly expressed toxins (> 50% of toxin transcripts) were the major components in each venom proteome (Fig. 1), and the primary lethal or tissue damaging toxins in these venoms [54, 65–69]. This analysis also validated the use of these genomes and *de novo* assembled transcriptomes, where even given these different approaches (e.g. genomes sequenced with different technologies and toxins annotated by different labs), we still found toxin expression correlated with the venom proteome for each species.

For toxin expression in the *P. textilis* MVG, highly abundant toxins (3FTx_1, 2, 3, 4 and PLA2_1) exhibited TPM increases (Fig. 1A) with overall fold-changes of two-fold or less in comparison to the UVG. There were small decreases in expression (less than 30%) for 5’NUC, two 3FTxs (3FTx_8 and 10), two PLA_2_ (PLA2_2 and 3) and HYAL, and larger decreases (36–66%) for two 3FTxs (3FTx_7 and 11), AChE, two CYSs (CYS_1 and 2), two KUN (KUN_1 and 2), two NPs, four snaclecs (SNAC_5, 8, 11, and 13) and WAP. Snaclec transcript isoforms exhibited the greatest variability between the MVG and UVG; SNAC_2 had the overall highest increase (11.4-fold) and SNAC_5 had the greatest decrease (0.31-fold).

For toxin expression in the viperid MVG, SVMP_1 in *C. viridis* was the top upregulated transcript by 43-, 58-, and 127-fold at 24, 72, and 96 hpvm, respectively. However, there were not parallel increases in expression for all rattlesnake toxins over the time course. For *C. viridis*, the highest expressed PLA_2_ (PLA2_A1) peaked 72 hpvm and another PLA_2_ (PLA2_B1), the majority of SVMPs (7 out of 11) and myotoxin_1 peaked at 96 hpvm (Fig. 1B). SVSP transcripts in *C. tigris* venom glands showed the greatest fold changes over the time course: SVSP_1 (XM_039325703.1) was upregulated 699-fold at 24 hpvm and SVSP_2 (XM_039360544.1) was the top upregulated toxin transcript (45-fold) at 96 hpvm.

### UPR, Notch signaling, and cholesterol homeostasis are enriched in MVGs, and different biological process regulated between *P. textilis* and viperids

A Gene Set Enrichment Analysis (GSEA) [71, 72] was conducted using human gene orthologs for all non-toxin genes expressed in MVGs and UVGs. No gene sets were found to be significantly enriched in MVGs for any of the three species, but the lowest familywise error rate (*p*-value = 0.50) was for UPR in *P. textilis*, Notch signaling in *C. viridis* (familywise error rate *p*-value = 0.49) and cholesterol homeostasis in *C. tigris* (familywise error rate *p*-value = 0.49), although these three biological processes were listed in enriched datasets of all three species with the exception of cholesterol homeostasis in *C. viridis* (Supplemental Table 8).

**Fig. 2:**
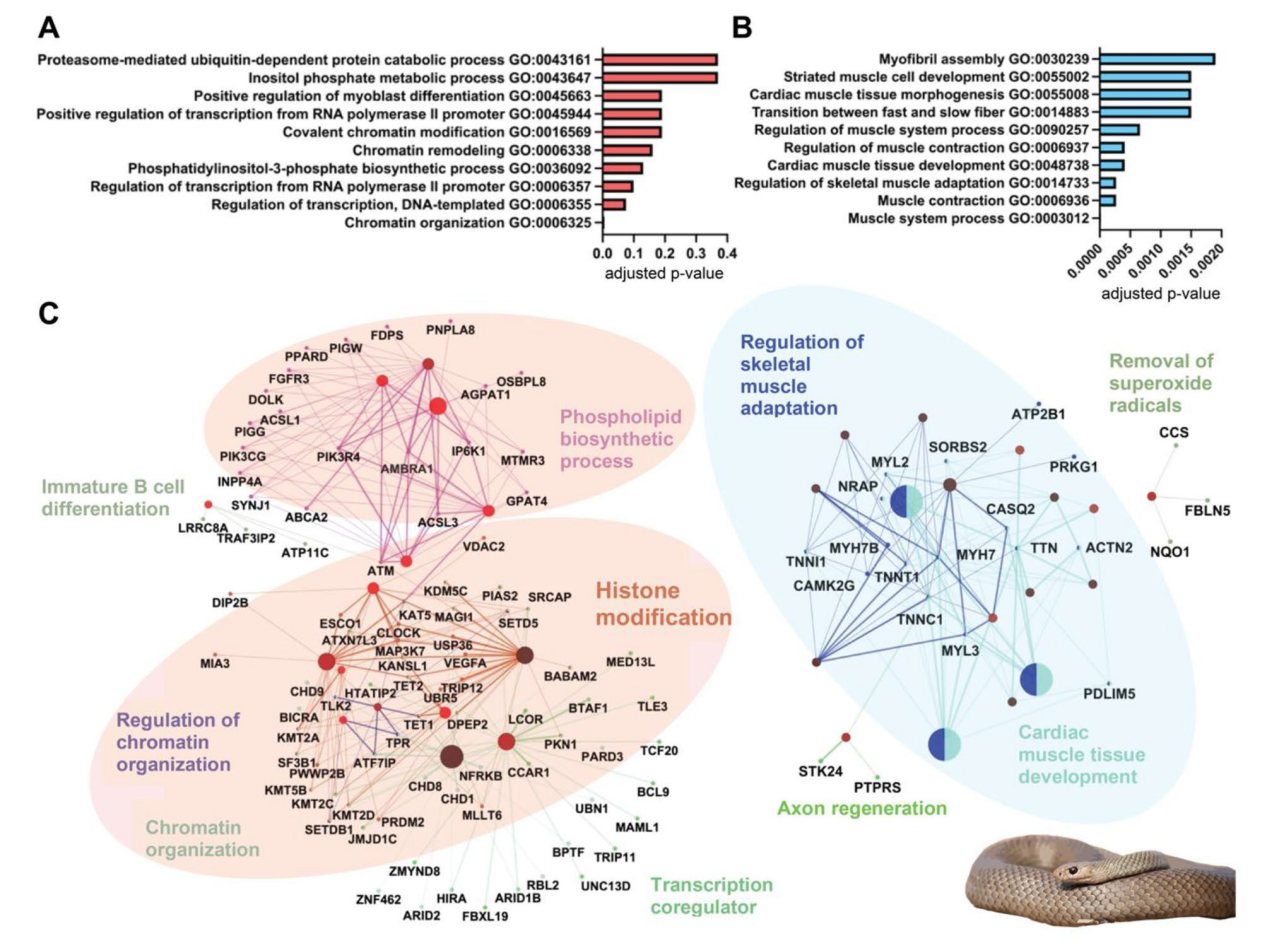
Enriched biological processes and associated networks for genes up- and downregulated in *Pseudonaja textilis* milked venom gland. The top ten biological processes are shown with their level of significance for genes (**A**) upregulated at least 10-fold and (**B**) downregulated to less than 0.10-fold 96 hpvm. Gene ontology analysis was completed using DAVID Bioinformatics Resources [73, 74] and Benjamini-Hochberg adjusted *p*-values were used for identifying levels of significance. For each up- and downregulated gene set, (**C**) gene networks and associated biological processes were generated using the ClueGo app plug-in [75] in Cytoscape [76] with *Homo sapiens* orthologs. A minimum number of 3 genes was required for pathway selection. Light red colored ovals highlight upregulated gene networks with the greatest number of related nodes, and the light-colored blue oval for those downregulated. Photo credit: *P. textilis*, Ákos Lumnitzer

Focusing on genes with the greatest fold-change between the MVG and UVG from *P. textilis*, we selected all genes upregulated at least 10-fold (373) in the MVG and those that exhibited less than a 0.1-fold difference (415) compared to the UVG. A gene ontology and network analysis of the upregulated gene set found the following overrepresented biological processes: chromatin and histone remodeling (GO:006325, GO:0016569, and GO:006338), regulation of transcription (GO:0006355, GO:0006357, and GO:0045944) and phospholipid biosynthesis (including phosphatidylinositol-3-phosphate signaling GO:0036092 and inositol phosphate metabolic process GO:0043647) (Fig. 2A,C). Chromatin organization was found to be the only significantly upregulated biological process (*p* = 0.0005; for details, see below). Downregulated genes were significantly enriched for proteins involved in striated muscle contraction and sarcomere assembly (*p* < 0.002; GO:0006936, GO:0014733, GO:0006937, GO:0090257, GO:0055002, and GO:0030239) (Fig. 2B,C).

**Fig. 3:**
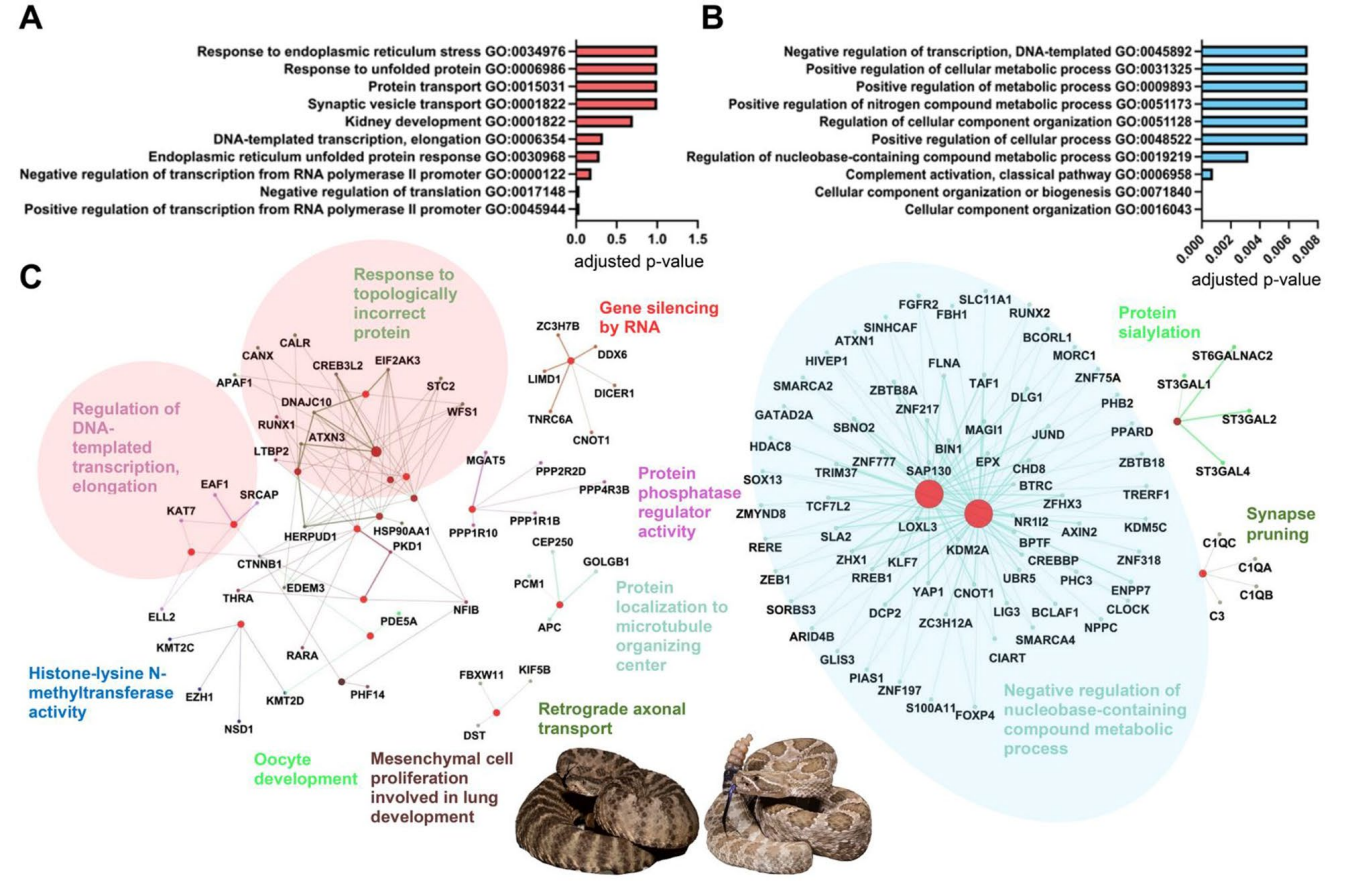
Enriched biological processes and associated networks for genes up- and downregulated after venom milking viperids *Crotalus viridis* and *C. tigris.* The top ten biological processes are shown with their level of significance for genes (**A**) upregulated at least 10-fold and (**B**) downregulated to less than 0.10-fold at 96 hpvm. Gene ontology analysis was completed using DAVID Bioinformatics Resources [73, 74] and Benjamini-Hochberg adjusted *p*-values were used for identifying levels of significance. For each up- and downregulated gene set, (**C**) gene networks and associated biological processes were generated using the ClueGo app plug-in [75] in Cytoscape [76] with *Homo sapiens* orthologs. A minimum number of 3 genes was required for pathway selection. Light red colored ovals highlight upregulated gene networks with the greatest number of related nodes, and the light-colored blue oval for those downregulated. Photo credits: *C. tigris*, Ben Lowe; *C. viridis*, Wolfgang Wüster

For the viperids *C. viridis* and *C. tigris*, 16 and 197 genes were upregulated, and 85 and 515 genes downregulated 96 hpvm, respectively, using the same thresholds as used for *P. textilis*. This was out of a total of 17,483 expressed transcripts for *C. viridis* and 58,086 transcripts for *C. tigris.* A gene ontology and network analysis of the upregulated gene set found the following overrepresented biological processes: transcription (GO:0045944, GO:0000122, and GO:006354), protein translation and transport (GO:0017148, GO0001822, and GO:0015031), and the UPR (GO:0030968) (Fig. 3A, C). Positive regulation of transcription and negative regulation of translation were significant upregulated biological processes (*p* < 0.05); Fig. 3A). Downregulated biological processes were complement activation (GO:0006958 and GO:0006956), immune response (GO:0006954, GO:0045087, and GO:0006955), cellular component organization (GO:0016043, GO:0071840, and GO:0051128) and metabolic processes (GO:0019219, GO:0051173, GO:0009893, and GO:0031325) (Fig. 3B); all were significant (*p* < 0.008). A gene regulatory network analysis of the downregulated gene sets found the largest gene network related to negative mechanisms of regulating nucleobase-containing macromolecules, which included transcriptional repressors (Fig. 3C).

### Chromatin remodelers and transcription factors are differentially regulated after venom miking between *Pseudonaja textilis* and viperids

Given the upregulation of genes involved in chromatin organization and histone modification in the *P. textilis* MVG (Fig. 2A, C), we manually identified all chromatin modifiers upregulated at least 40-fold: Snf2 related CREB activator protein (*SRCAP*, 86-fold), jumonji domain containing 1 C (*JMJD1C*, 43-fold), lysine methyltransferases 2A (*KMT2A*, 80-fold), KMT2C (*KMT2C*, 45-fold), KMT2D (*KMT2D*, 64-fold), and chromodomain-helicase-DNA-binding protein 8-like (*CHD8*, 52-fold) (Table 1 and Supplemental Table 9). *CHD8* was upregulated only 2-fold in the *C. viridis* MVG at 72 hpvm and *SRCAP, KMT2C* and *KMT2D* were upregulated 12-, 24- and 18-fold, respectively, in the *C. tigris* MVG at 96 hpvm. Chromatin modifiers were not seen as highly upregulated in viperid MVGs (Supplemental Table 10).

**Table 1:** Gene expression and activities of upregulated chromatin remodeler genes and transcription factors in elapid and viperid milked venom glands

**Chromatin remodeler genes**	**Elapid 96 hpvm**	**Viperids 96 hpvm**	**Activity**
*SRCAP*	86-fold	*C. viridis*: 3-fold *C. tigris*: 12-fold	A coactivator for several TFs (CREB, the glucocorticoid receptor and the androgen receptor), and functions by exchanging nucleosome histones to render DNA accessible for transcription [77–79].
*JMJD1C*	43-fold	*C. viridis*: N.C. *C. tigris*: 3-fold	Alters chromatin accessibility by histone demethylation, activating transcription [80].
*KMT2A, KMT2C*, and *KMT2D*	80-, 45-, and 64-fold	*C. viridis*: 1-, N.C., and 2-fold *C. tigris*: 2-, 24-, and 18-fold	Methylate lysine 4 of histone 3 (H3), a tag for epigenetic transcriptional activation [81–83].
*CHD8*	52-fold	*C. viridis*: N.C. *C. tigris*: 5-fold	Interacts with H3 di- and tri-methylated at lysine 4, recruiting histone H1 and repressing genes regulated by β-catenin (Wnt signaling pathway) [84–86].
**Transcription factor genes**			**Activity**
*SP1*	79-fold	*C. viridis*: 4-fold *C. tigris*: 5-fold	Binds GC box and related GT/CACC box regulatory elements in promoter, enhancer and locus control regions of housekeeping and tissue-specific genes [87].
*FOXN2*	41-fold	*C. viridis*: N.C. *C. tigris*: 12-fold	Binds purine-rich regions and members of this TF family have been implicated as regulators of embryogenesis, cell cycling, and cell lineage restriction [88].
*LCOR*	40-fold	*C. viridis*: N.A. *C. tigris*: 2-fold	Can function as either a transcription activator or repressor and has been linked to polycomb-group target genes, promoting methyltransferase activity [89, 90].
*CREB3L3*	N.C.	*C. viridis*: 11-fold *C. tigris*: N.A.	TF involved in ER stress and activating the unfolded protein response [91].
*NFIA, NFIB*, and *NFIX*	17-, 5-, and 17-fold	*C. viridis*: 5-, 6-fold, and N.C. *C. tigris*: 31-, 133-, and 24-fold	NFI family of TFs regulates genes across many different cell types, recognizing a palindromic consensus DNA sequence TGGA/C(N)_5_GCCAA [92].

N.C. = No Change; N.A. = Not Annotated

Although chromatin structure regulates the accessibility of gene regulatory elements, TFs play vital roles in the regulation of transcription. The TF with the greatest fold upregulation in the *P. textilis* MVG, and with a transcript variant X2 uniquely expressed in the MVG, was specificity protein 1 (*SP1*, 79-fold) (Table 1 and Supplemental Table 9). In the MVG of the rattlesnakes *C. viridis* and *C. tigris*, *SP1* was only slightly upregulated comparatively, 1.5-, 2- and 4-fold at 24, 72 and 96 hpvm in *C. viridis* and 14- and 5-fold at 24 and 96 hpvm, respectively, in *C. tigris.* In addition, Forkhead box N2 (*FOXN2*) and ligand-dependent corepressor (*LCOR*) were upregulated 41- and 40-fold, respectively, in the *P. textilis* MVG (Supplemental Table 9), but not to this extent in either viperid (Table 1). The TF with the greatest fold-change in the *C. viridis* MVG was cAMP-responsive element binding protein 3-like (*CREB3L3*), which was upregulated 14-, 12-, and 11-fold at 24, 72, and 96 hpvm, respectively. Nuclear factor I isoforms (*NFIA, NFIB*, and *NFIX*) were found to be the TFs with the greatest upregulation in *C. tigris*. *NFIA* was upregulated 31-fold at 96 hpvm, *NFIB* was upregulated 104-fold and 133-fold, and *NFIX* was upregulated 8- and 24-fold at 24 hpvm and 96 hpvm, respectively, in *C. tigris* (Table 1 and Supplemental Table 10). *NFIA* and *NFIB* were also found upregulated 5-fold and 6-fold, respectively, at 96 hpvm in *C. viridis* (Table 1).

### *Cis*-regulatory elements (CREs) and *trans-*factor upregulation vary between *Pseudonaja textilis* and viperids

**Fig. 4:**
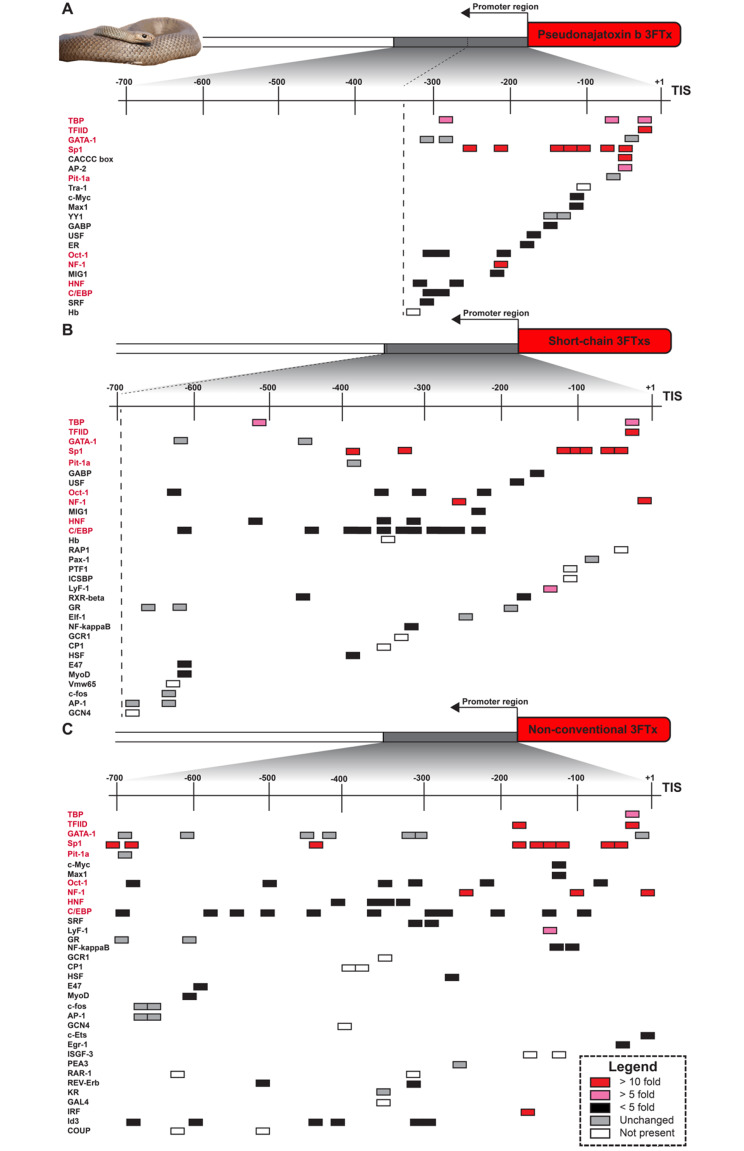
Predicted *cis-*regulatory elements in the promoter regions of *Pseudonaja textilis* three-finger toxins. Only the first 341 bp upstream from the Transcription Initiation Site (TIS) could be evaluated for (**A**) three-finger toxin (3FTx) pseudonajatoxin b (AY027493) and 684 bp upstream from the TIS for (**B**) short-chain 3FTxs (AF204969) due to limited sequence availability and fragmented genome assembly. *Pseudonaja textilis* did not express transcripts encoding non-conventional 3FTxs in the venom gland, despite the presence of a non-conventional 3FTx gene in its genome (XP_026561523). From the genome reference, 713 bp upstream from what would be the TIS for this (**C**) non-conventional 3FTx was evaluated. Fold-changes in expression levels are shown between the *P. textilis* milked venom gland and unmilked venom gland for *trans*-factors known to interact with predicted *cis-*regulatory elements (CREs). CRE predictions were completed with the online server AliBaba2.1 using the TRANSFAC 4.0 database. CREs shared across panels are bolded red. Photo credit: *P. textilis*, Ákos Lumnitzer

Using toxin gene promoter regions from elapids and viperids, we predicted CREs and evaluated the expression of corresponding *trans*-regulatory factors in MVGs. Promoter activities determined either by reporter gene chloramphenicol acetyl transferase or luciferase assays have identified the importance of CREs within the first 500 base pairs upstream of toxin genes [38, 40]. Due to variability in toxin gene expression, we evaluated regions upstream each toxin gene isoform independently to determine CREs potentially contributing to differential expression. For *P. textilis*, this included 341 bp for the 3FTx pseudonajatoxin b (AY027493) (Fig. 4A), 684 bp for short-chain 3FTx (AF204969) (Fig. 4B), and 714 bp for a non-conventional 3FTx (Fig. 4C). Only one gene is present for pseudonajatoxin b [93], and five different short-chain 3FTx genes share the same 684 bp promoter sequence [94]. Interestingly, among 3FTxs, although many Old World elapids have non-conventional 3FTxs in their venoms with a fifth disulfide bond in the first loop [95], *P. textilis* did not express non-conventional 3FTxs in the venom gland, despite the presence of a non-conventional 3FTx gene in its genome (XP_026561523). We included this toxin gene in the analysis to determine if unique CREs were present for a non-expressed 3FTx.

Predicted CREs in promoter regions of the three 3FTx classes varied, likely due to nucleotide sequence diversity, as there was 86–88% conserved sequence in the first 341 bp; however, we did identify shared sites for TATA-box binding proteins (TBP) and TATA-box binding protein associated factors of the RNA polymerase II preinitiation complex (TFIID). Additionally, there were GATA-1, Sp1, Pit-1a, Oct-1, NFI, HNF, and c/EBP binding sites across all promoters, but these varied in number and location. All 3FTx gene promoter sequences did have multiple (six to nine) Sp1 binding sites (Fig. 4). Of the predicted TFs binding to 3FTx gene promoter regions, Sp1/CACCC-box, NFI, and interferon regulatory factor (IRF) were found upregulated at least 10-fold in the *P. textilis* MVG. The IRF binding site was only present in the non-conventional 3FTx gene promoter sequence. We did not find any TFs that were downregulated to less than 0.10-fold, which was our threshold.

Non-conventional 3FTxs or plesiotypic 3FTx-like homologs are also present in viperid genomes [96], although these toxin genes are usually either absent or lowly expressed in viperid venom glands [97]. To compare CRE predictions for non-conventional 3FTxs in both elapid and viperids, we identified 700 bp regions upstream of the closest *P. textilis* non-conventional 3FTx (XP_026561523) homologs in *C. viridis* and *C. tigris* (Supplemental Fig. 3). The closest 3FTx homolog in *C. tigris* (LOC120302985) exhibited a gene promoter region with more similarity to that of the *P. textilis* non-conventional 3FTx (XP_026561523) than *C. viridis;* however, GATA-1, Pit-1a, Oct-1 sites were positions farther upstream, and there was unchanged or reduced expression of these *trans*-factors at 96 hpvm.

**Fig. 5:**
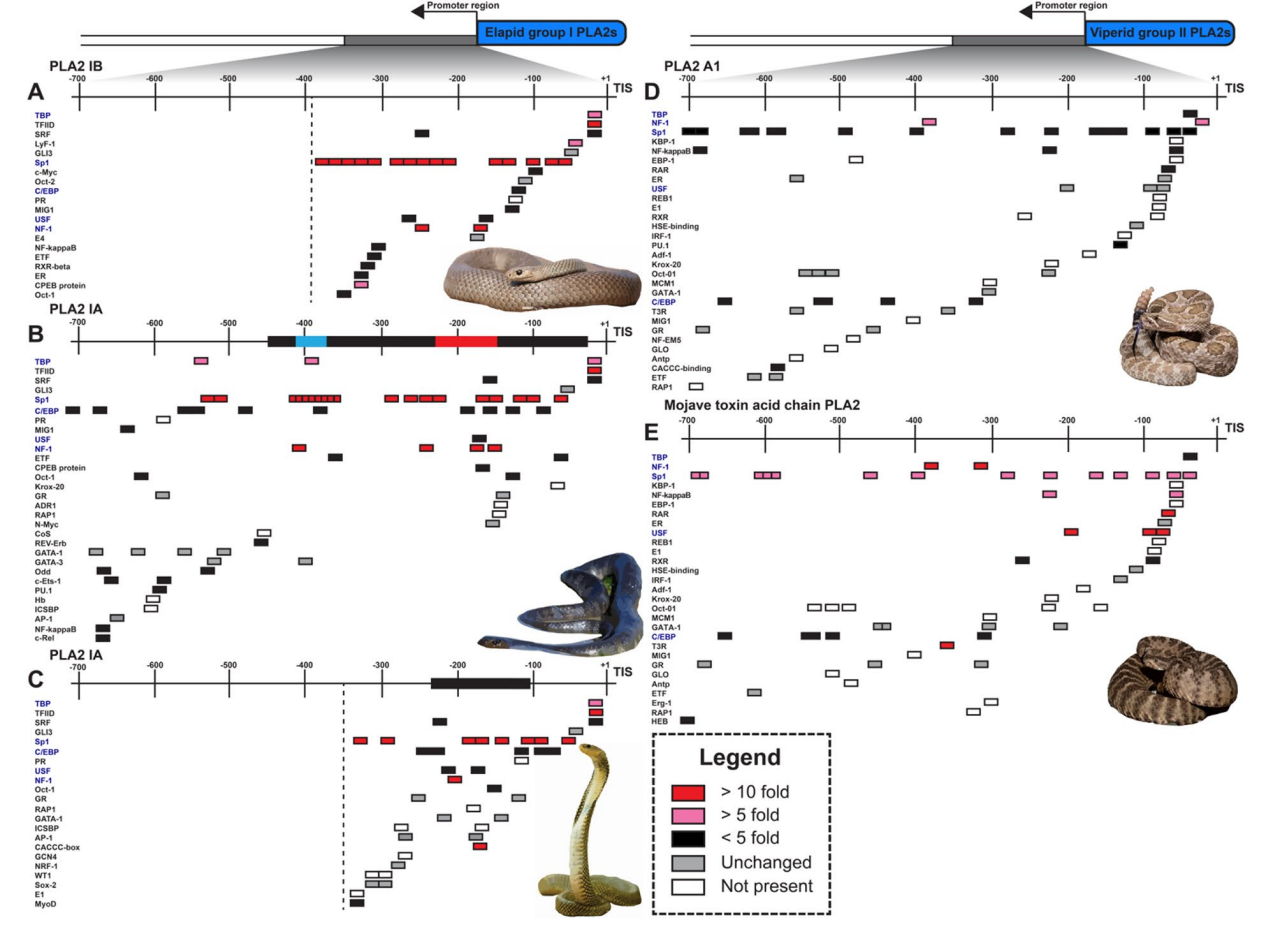
Predicted *cis-*regulatory elements in promoter regions of highly expressed group I and group II phospholipase A_2_s. Promoters regions upstream from Transcription Initiation Sites (TISs) are shown: 385 bp for (**A**) group IB PLA_2_ (AY027495) from *Pseudonaja textilis*, 706 bp for (**B**) group IA PLA_2_ (AB111958) from *Laticauda semifasciata*, 367 bp for (**C**) group IA PLA_2_ (AF101235) from *Naja sputatrix*, 702 bp for (**D**) group II PLA_2__A1 from *Crotalus viridis*, and 705 bp for (**E**) group II PLA_2__acidic (XM_039367474) from *C. tigris.* Fold-changes in expression levels are shown between milked and unmilked glands (96 h post venom milking) for *trans*-factors known to interact with the predicted *cis-*regulatory elements (CREs). Identified regulatory regions are bolded in promoter regions for AB111958 [98] and AF101235 [38]. This includes a region from − 232 to -162 in AB111958 that was found to be responsible for an increase in promoter activity (bolded red) and a suppressor region from − 410 to -382 (bolded blue) [98]. CRE predictions were completed with the online server AliBaba2.1 using the TRANSFAC 4.0 database. CREs shared across panels are bolded blue. Photo credits: *P. textilis*, Ákos Lumnitzer; *L. semifasciata*, Patrick Davis; *N. sputatrix*, Matej Dolinay; *C. viridis*, Wolfgang Wüster; *C. tigris*, Ben Lowe

CREs upstream of PLA_2_ genes that are highly expressed in elapids (group I PLA_2_) and viperids (group II PLA_2_) were also evaluated (Fig. 5). Elapid group I PLA_2_s are subdivided into group IA and IB, group IB is likely the ancestral PLA_2_ gene with the presence of the complete pancreatic loop [98]. For *P. textilis*, group IB PLA_2_s are the most abundant and two group IB PLA_2_ genes have been found in the *P. textilis* genome with identical 385 bp sequence upstream from the transcription initiation site (TIS) (Fig. 5A) [99]. For *Laticauda semifasciata* and *Naja sputatrix*, group IA PLA_2_s are the most abundant, and 706 bp and 367 bp upstream from TISs were evaluated, respectively (Fig. 5B,C) [38, 98]. Group II PLA_2_s with the highest expression levels in *C. viridis* and *C. tigris* venom glands were PLA2_A1 and PLA2_acidic (XM_039367474), respectively, and just over 700 bp of promoter regions were evaluated for each (Supplemental Tables 5 and 6, Fig. 5D,E). Regardless of group IA, IB or II, CREs for binding TBP, Sp1, c/EBP, USF, and NFI were present for all expressed PLA_2_ genes. Multiple Sp1 binding sites (eight to 19) were observed clustered together within all PLA_2_ upstream gene regions. For the *P. textilis* group IB PLA_2_, TFs with CREs present and upregulated at least 10-fold in the MVG were Sp1/CACCC-box and NFI, the same two TFs as seen for 3FTxs. For group II PLA_2_s in the viperids, TFs with CREs and upregulated over 10-fold were NFI, retinoic acid receptor (RAR), upstream stimulatory factor 1 (USF1), and thyroid hormone (3,5,3’-triiodothyronine) receptor (T3R) (Fig. 5D,E). Interestingly, although Sp1 was slightly upregulated (5-fold) in *C. tigris* 96 hpvm, this was not observed for *C. viridis*, but NFI was upregulated at least 5-fold in both viperids. TFs that were downregulated less than 0.10-fold did not have any predicted CREs.

Group I PLA_2_ homologs are also present in viperid genomes but are not biological relevant toxins for this snake family [27]. For comparisons between group I PLA_2_ toxin gene promoter regions and that of non-toxin group I PLA_2_ in viperids, we identified 700 bp regions upstream of the closest *P. textilis* group IB PLA_2_ (AY027495) homologs in *C. viridis* and *C. tigris* (Supplemental Fig. 4). There were 15 predicted Sp1 binding sites for the group IB PLA_2_ toxin gene in *P. textilis*, but only five and seven binding sites for the non-toxin group I PLA_2_ in *C. viridis* and *C. tigris*, respectively. There was an abundance, up to 16, of c/EBP binding sites, and an absence of USF binding sites in the promoter regions of non-toxin group I PLA_2_s in *C. viridis* and *C. tigris.* NFI binding sites were also found to be quite variable between group I and group II PLA_2_s in *C. viridis* and *C. tigris*.

Additionally, we evaluated upstream regions that have been experimentally shown to regulate PLA_2_ toxin gene promoter activity. Fujimi et al. (2004) found a 411 bp insertion sequence (-444 to -34) present in the highly expressed group IA PLA_2_s that was absent in the lowly expressed group IB for *L. semifasciata*. Luciferase activity assays from construct variations of this insertion identified a region from − 232 to -162 that triggered elevated expression and a suppressor region from − 410 to -382 [98]. Jeyaseelan et al.(2000) used chloramphenicol acetyl transferase reporter gene assays and DNase 1 footprinting approaches with promoter constructs from a *N. sputatrix* group I PLA_2_ gene to identify a region from − 116 to -233 that contained crucial CREs [38]. We found CREs with binding sites for Sp1 and NFI in all of these identified regulatory regions (Fig. 5B, C). Previously, we identified a 271 bp insertion (-308 to -37) upstream of the gene for pseutarin C catalytic subunit (venom coagulation factor X) that differed from the endogenous coagulation factor X gene. We termed this segment *VERSE* (*Ve*nom *R*ecruitment/*S*witch *E*lement) [100]. Within the *VERSE* core promoter there are two regions that upregulate the pseutarin C catalytic subunit (Up1 and Up2) and one that suppresses expression (Sup1) [40]. Here, we re-analyzed the TFs binding to these regulatory regions and found CREs for Sp1 in Up2 and c/EBPdelta in Sup1 (Supplemental Fig. 5). c/EBPdelta was downregulated 0.82-fold in the *P. textilis* MVG.

### Venom gland miRNA profiles and targets are distinct between elapid and viperid snakes, and after venom milking

MiRNAs are known to post-transcriptionally regulate over 60% of mammalian genes [101]. We sequenced small RNA-seq libraries from the *P. textilis* MVG and UVG, and MVGs from *C. viridis* (96 hpvm), to examine miRNA expression and regulation in snake venom glands. A total of 366 miRNAs (308 non-redundant mature sequences) in the MVG and 375 miRNAs (299 non-redundant mature sequences) in the UVG were identified in *P. textilis* (Supplemental Table 11), and 501 miRNAs (420 non-redundant mature sequences) were identified in the MVGs from *C. viridis* (Supplemental Table 12). Using information from the chromosome-level *C. viridis* genome assembly, we found the greatest numbers of miRNA genes present on chromosomes 2 (96 genes), 1 (94 genes), and 3 (64 genes), whereas the other smaller macrochromosomes and microchromosomes had less (< 30 genes). Approximately 50% of miRNAs from the *P. textilis* MVG and UVG were common (Supplemental Fig. 6A), but only 18% of miRNAs found in *C. viridis* were also present in *P. textilis* (Supplemental Fig. 6B). The most abundant miRNAs in *P. textilis* venom glands were found to be *miR-148a-3p* with 359,436 Counts Per Million reads (CPM) in the MVG and *miR-10c* with 210,834 CPM in the UVG (Supplemental Fig. 7A, B). *miR-375* had been found to be the most abundant miRNA in the venom gland of the king cobra (*Ophiophagus hannah*) [102] (Supplemental Fig. 7C), but this was not the case for *P. textilis* where it was ranked 13th in the MVG and 7th in the UVG. Interestingly, *miR-375* was not present in the venom gland of *C. viridis.* For *C. viridis, miR-21-5p* was the most abundant (157,080 CPM), followed by *miR-148a-3p* (140,288 CPM) (Supplemental Fig. 7D), both of which were present but varied in expression between the elapid and viperid venom glands (Table 2).

**Table 2:** miRNA differences between elapid and viperid milked venom glands

miRNA	Elapid 96 hpvm	Viperid 96 hpvm	Activity
*miR-375*	Present	Absent	Multiple transcript targets predicted that relate to ubiquitin regulation, stress response, collagen biosynthesis, translation, endocytosis and trafficking (Supplemental Table 14) [103].
*miR-148a-3p*	359,436 CPM	140,288 CPM	Predicted to target C-type lectin-like toxin transcript in *P. textilis* (SNAC_5), no predicted targets in viperids (Supplemental Tables 13 and Supplemental Table 15).
*miR-21-5p*	22,809 CPM	157,080 CPM	No predicted mRNA targets in the elapid but predicted to target the N-alpha-acetyltransferase 25 auxiliary subunit in viperid (Supplemental Table 15).
*miR-215-5p*	Absent	Present	Predicted to target a SVMP transcript (SVMP_4) in *C. viridis* MVGs (Supplemental Table 13).
Top ten miRNAs	Toxin transcripts targeted: Snaclecs, group I PLA_2_s, 3FTxs and pseutarin C.	Toxin transcripts targeted: SVMPs	Different toxin transcript targets were predicted from elapid and viperid venom glands (Supplemental Table 13).
miRNAs over 100 CPM	mRNA transcripts targeted: Intracellular transport, catabolic processes, metabolic processes, organelle organization, and ER to Golgi mediated transport.	mRNA transcripts targeted: Regulation of mRNA processing, catabolic processes, negative regulation of cytoskeleton organization, steroid hormone receptor signal, stress granule assembly, transport, and ER stress.	Transcripts for proteins involved in different biological processes were targeted in elapid and viperid MVGs (Fig. 6).
*Pte-miR-1*	Present in the *P. textilis* MVG	Absent	Predicted to target 510 mRNAs expressed in the *P. textilis* MVG. These included transcripts for proteins involved in ER to Golgi vesicle transport, ubiquitin-dependent ERAD pathway and proteasome-mediated ubiquitin-dependent protein catabolic process (Supplemental Fig. 8).

miRNAs can post-transcriptionally repress or enhance mRNA translation by base pair complementary binding [104], usually within the 3’ untranslated region (3’ UTR) of target mRNAs, but also within the 5’ UTR or coding sequence [105]. To explore potential miRNA regulation of toxin transcripts [106], toxin transcript targets were predicted for the top ten most abundant miRNAs. In the *P. textilis* MVG, miRNAs targeted transcripts for seven C-type lectin-like toxins (SNAC_2, 3, 5, 7, 8, 9, and 12), all three PLA_2_s, and the pseutarin C catalytic subunit. In the *P. textilis* UVG, the same toxin transcripts were targeted with an additional six short-chain 3FTxs (3FTx_7, 8, 10, 11, 12 and 13) and the pseutarin C non-catalytic subunit (Table 2). In the *C. viridis* venom gland, two snake venom metalloprotease transcripts (SVMP_4 and 9) were targeted (Supplemental Table 13), although not all toxin transcripts were evaluated (only those listed in Supplemental Table 5). *miR-215-5p*, which targeted the SVMP_4 transcript, was found uniquely expressed in the *C. viridis* venom gland (Table 2 and Supplemental Fig. 7D).

**Fig. 6:**
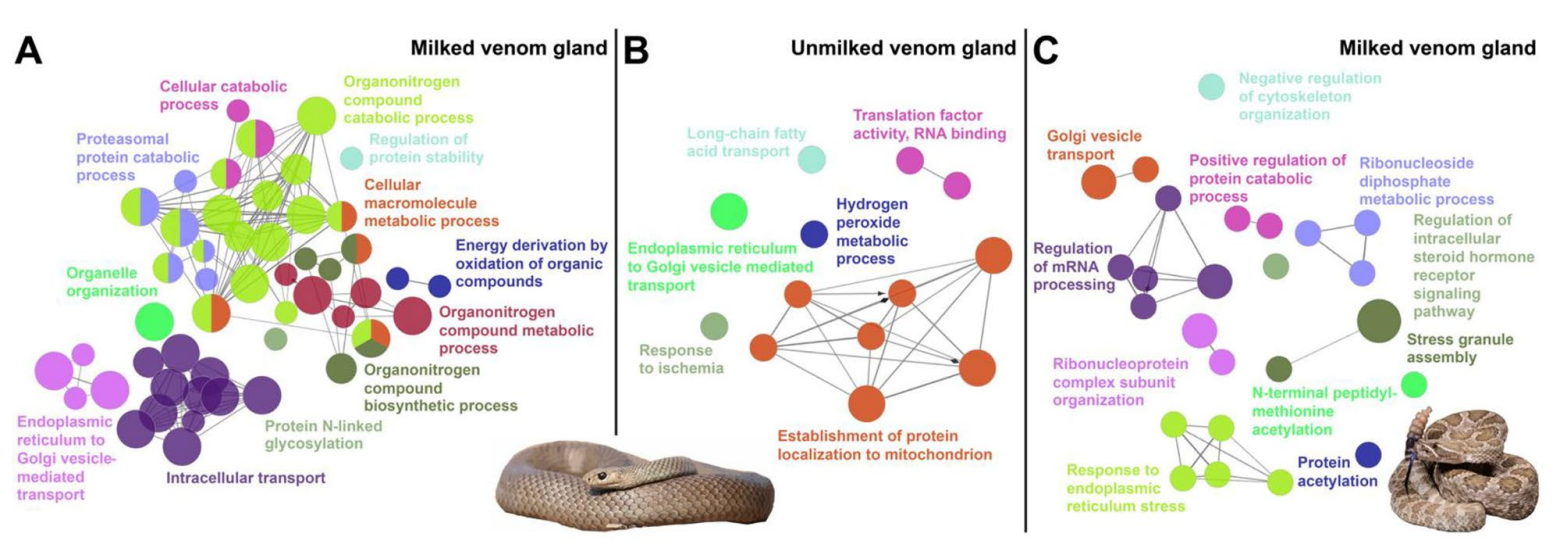
Gene networks and enriched biological processes for transcripts that are targeted by miRNAs expressed in snake venom glands. Transcripts from *Pseudonaja textilis* and *Crotalus viridis* genome annotations were used to predict mRNAs targeted by abundant miRNAs (over 100 Counts Per Million) in the (**A**) milked venom gland from *P. textilis*, (**B**) unmilked venom gland from *P. textilis*, and (**C**) milked venom glands from *C. viridis.* Gene networks were generated using *Homo sapiens* ortholog accessions of all targeted transcripts and the ClueGo app plug-in [75] in Cytoscape [76] to visual significant associated biological processes. Photo credit: *P. textilis*, Ákos Lumnitzer; *C. viridis*, Wolfgang Wüster

Next, we predicted mRNAs targeted by abundant miRNAs (over 100 CPM) using all transcript annotations from the *P. textilis* and *C. viridis* genomes to identify the most likely regulated mRNAs and pathways. A total of 750, 264, and 244 transcripts in the *P. textilis* MVG, *P. textilis* UVG and *C. viridis* MVGs, respectively, met these criteria with predicted miRNA binding sites (Supplemental Tables 14 and 15). Gene regulatory network analyses of miRNA targets identified a greater number of biological processes targeted in MVGs, especially for *P. textilis* (Fig. 6). Biological processes targeted in *P. textilis* MVG included intracellular transport (GO:0006886 and GO:0006888) and several metabolic and catabolic related processes (GO:0043170, GO:1,901,565, GO:1,901,564, among others) (Fig. 6A). With the exception of ER to Golgi vesicle transport, different biological processes were targeted in the *P. textilis* UVG (Fig. 6B). Targeted processes in *C. viridis* MVGs shared interestingly little overlap to those of *P. textilis* and included regulation of mRNA processing (GO:0006397), response to endoplasmic reticulum stress (GO:0034976), and ribonucleoside diphosphate metabolic process (GO:0009185), among others (Fig. 6C; Table 2).

We observed transcripts with multiple miRNA binding sites, as well as miRNAs that targeted multiple transcripts. One novel miRNA (3’-cgCCGCCGCCGTCGCCGCc-5’), uniquely expressed in the *P. textilis* MVG, had 510 targets. This miRNA did not share identity to any currently in the miRBase database [107], and due to the potential importance of this miRNA, we have named it *Pte-miR-1*. It is located on the *P. textilis* genome scaffold NW_020769308.1, 14,222,810 bp to 14,222,853 bp in the sense direction. Transcripts targeted by *Pte-miR-1* were for proteins involved in the following biological processes: ER to Golgi vesicle transport (GO:0006888), ubiquitin-dependent ERAD pathway (GO:0030433) and proteasome-mediated ubiquitin-dependent protein catabolic process (GO:0043161) (*p*-value = 0.008) (Supplemental Fig. 8).

## Discussion

Snakes of the families Elapidae and Viperidae are responsible for medically relevant snakebite envenoming worldwide, and venom variation between and within these families impacts the management of snakebite victims [15, 108]. Investigations into how toxin genes, which most commonly arise from gene duplications, are variably expressed have been few due to the paucity of non-model genomes, including those for venomous snake species, and this question has been almost entirely unexplored for elapids. Using high throughput RNA-sequencing of MVGs and UVGs from the elapid *P. textilis* and viperids *C. viridis* and *C. tigris*, as well as comparative genomic approaches evaluating CREs in promoter regions of highly expressed toxin genes, we identified distinct toxin gene regulatory networks between *P. textilis* and the two viperids.

3FTxs, group I PLA_2_s, KUNs, Snaclecs, and pseutarin C were the most abundant toxin families expressed in *P. textilis* venom glands (Fig. 1A). Geographic variation in venom composition is found between South Australian (SA) and Queensland (QLD) *P. textilis* populations; SA snakes have an abundance of postsynaptic neurotoxins (3FTxs) and QLD snakes have greater amounts of presynaptic textilotoxin and procoagulant pseutarin C in their venoms [58]. We previously evaluated the venom proteomes of 12 *P. textilis* individuals from SA and found none contained all textilotoxin subunits, three individuals even entirely lacked textilotoxin subunits in their venoms [54], but it was uncertain if this was due to method sensitivity limitations. Our high-throughput transcriptomic results from a *P. textilis* SA individual demonstrates a complete lack of gene expression of all four textilotoxin PLA_2_ subunits, and pseutarin C expression was only 2% of total toxin transcripts, whereas 3FTxs were highly expressed, exhibiting greater than 70% of total toxin transcription. These data demonstrate that *P. textilis* venom variation is partly due to differences in toxin gene transcription between populations. Evaluation of *P. textilis* genomes from different geographic regions would provide insight into resolving whether PLA_2_ genes for textilotoxin subunits are present but lacking transcription, or if these genes have been lost altogether in certain populations. This could contribute to the noted ‘brown snake paradox’, where although textilotoxin is a potent neurotoxin in *P. textilis* venom, *P. textilis* envenoming more frequency causes coagulopathy disturbances and rarely neurotoxicity [109].

Toxin expression in the viperid venom glands consisted primarily of myotoxins, SVSPs, group II PLA_2_s, and SVMPs for *C. viridis*, and group II PLA_2_s, SVSPs, BPPs, and VEGF for *C. tigris* (Fig. 1B, C). From 0 to 96 hpvm, there was asynchrony in toxin synthesis, corroborating observations for the Palestine Viper (*Daboia palaestinae*) [110]. However, different individual snakes were used at each time point for the viperid venom glands and intraspecific variation is likely. For this reason, we used a single *P. textilis* individual to investigate elapid toxin gene expression dynamics after venom milking.

Using MVGs and UVGs, we compared toxin gene expression between an elapid and viperids. Highly expressed *P. textilis* toxins exhibited fold-changes that were two-fold or less after venom milking. There were similar increases of 2.5–3.5-fold for 3FTxs and group I PLA_2_ genes 96 hpvm the venom gland of another elapid, *N. sputatrix* [23]. Increases over 40-fold for toxin gene expression were observed for the viperids *C. viridis* and *C. tigris*, with a SVSP gene peaking as high as 699-fold in *C. tigris* at 24 hpvm. Similar fold changes in SVSPs and SVMPs (over 50-fold and almost 20-fold, respectively) have been documented for the Puff Adder (*Bitis arietans*), although these levels of expression were determined from mRNA in venom, not venom glands [36]. The lower fold-changes in toxin gene expression in the elapid MVG is due to the high levels of toxin expression in the UVG, suggesting elapid toxin genes might be more constitutively expressed. Greater fold changes in viperid toxin expression after venom milking is also likely because of the extensive physiological changes (cell elongation and expansion of ER) that take place in secretory cells of viperid MVGs [24, 25], resulting in increased toxin synthesis capabilities. These physiological cell changes in viperid MVGs are largely absent in elapid MVGs [22, 23]. This also explains the absence of downregulated cellular component biogenesis and metabolic processes in the *P. textilis* MVG that was present in the viperid MVGs, which is likely due to the beginning downregulation of these extensive physiological processes that have been activated in viperids to account for the increased and peaking toxin production by this time point (Figs. 2B and 3B and B). Genes involved in striated muscle contraction were downregulated in the *P. textilis* MVG, aligning with the observation of the downregulation of troponin that was seen after venom milking of another elapid, the Many-banded Krait (*Bungarus multicinctus*) [111].

Genes related to the UPR, Notch signaling, and cholesterol homeostasis were enriched in the MVGs of both *P. textilis* and the two viperids. Increases in toxin synthesis, secretion, and posttranslational folding likely trigger upregulation of UPR and similar ER pathways to mediate ER stress and ensure protein quality control. Zhang et al. (2022) also found from a weighted gene co-expression network analysis an enrichment in the venom gland module of the elapid *B. multicinctus* for genes associated with protein folding and cytoplasmic transportation [9]. Perry et al. (2020) identified the UPR pathway as a feedback regulatory mechanism increasing venom production in the viperid *C. viridis* [112], and UPR pathway components were present in the conserved metavenom network of venom glands from the viper *Protobothrops mucrosquamatus* [113], as well as conserved across venom glands in Metazoa [114]. Cell-surface receptor Notch signaling is important in cell division and development [115], and Notch signaling has been found to be critical for salivary gland cell growth and differentiation [116]. In human salivary glands, β−adrenergic receptor activation upregulates Notch-mediated cell proliferation and differentiation of acinar cells [117], and this could be mediated by β-adrenergic receptor activation in the initiation of toxin synthesis in venom glands [42]. This additionally supports conserved higher-level regulatory networks between venom glands and salivary glands [113]. Cholesterol homeostasis enrichment is likely due to increases in vesicle-mediated transport and exocytosis in MVGs. Lipids that include cholesterol, phosphatidylinositol 4,5-bisphosphate and sphingolipids cluster as plasma membrane microdomains, concentrating and regulating SNARE proteins to create active exocytotic sites [118]. However, it should be noted that for our analysis, gene ontology networks were based on *Homo sapiens* orthologs, which do not necessitate a similar function in serpents.

We observed differences in chromosomal, TF, and CRE regulation between elapid- and viperid-specific venom production. Genes involved in chromatin organization/regulation, histone modification and transcription were upregulated in the *P. textilis* MVG (Fig. 2A, C). This included chromatin-remodeler *SRCAP* and histone lysine methyltransferases *KMT2A, KMT2C* and *KMT2D*. KMT2C and KMT2D, which are known to function together as super-enhancers [119], and could be a potential mechanism to increase transcription related to venom production. Although *SRCAP, KMT2C* and *KMT2D* were also upregulated in *C. tigris* after venom milking, there were less genes overall associated with chromatin organization/regulation upregulated for viperid MVGs (Fig. 3C). This could be due to the differences in chromosome locations of major toxins between elapids and viperids, macrochromosomes in elapids [9, 30] and microchromosomes in viperid snakes [31–33].

The transcription factor Sp1 was upregulated 79-fold in the *P. textilis* MVG, and Sp1 binding sites have been identified in promoter regions of *P. textilis* toxin genes for 3FTxs [93], group I PLA_2_s [99] and the pseutarin C catalytic subunit [100], as well as toxin genes from other elapids (Supplemental Table 16). We also identified clusters of Sp1 CREs in toxin gene promoters belonging to different venom protein families and isoforms (Figs. 4 and 5), and although Sp1 binding sites were also present in viperid toxin protomers and this TF was identified associated with super-enhancer regions for the viperid *C. viridis* [45], Sp1 was not upregulated to the same extent as observed for *P. textilis*, which corroborated previous work with *C. viridis* [45]. Additionally, there were fewer (5 or 6) Sp1 binding sites for the group I PLA_2_ homologs in *C. viridis* and *C. tigris* in comparison to the at least 8 binding sites seen for group I PLA_2_ genes in elapids. The shared Sp1 CREs of elapid 3FTx and group I PLA_2_ genes could allow for coordinated co-expression of these two venom protein families, which are both abundant toxins in the venom of many elapid snakes [27], and for a potential pre-adaptive regulatory mechanism that could have contributed to the evolution of defensive venom in spitting cobras [120]. Notably, Sp1 sites have been identified in regulatory promoter regions for 3FTx and group I PLA_2_ genes in the spitting cobra *N. sputatrix* [37, 38]. In *P. textilis*, shared Sp1 regulation of 3FTx, group I PLA_2_, and pseutarin C catalytic subunit genes could provide collective polygenic upregulation after venom milking.

We identified potential *cis-*regulatory suppressors of *P. textilis* toxin genes: the non-conventional 3FTx found in the *P. textilis* genome had an IRF binding site not found to be present in expressed 3FTx homologs, and a c/EBP binding site was identified within the suppressor region of the pseutarin C catalytic subunit gene. Further, c/EBP was downregulated 0.82-fold in the *P. textilis* MVG. Gamma interferon response elements (γ−IRE) contributed to cell-specific silencing of group I PLA_2_ genes in *N. sputatrix* [38], and c/EBP has been previously identified as a toxin suppressor CRE, as deletion of this site resulted in a 2-fold increase in promoter activity for a cytotoxic 3FTx in *N. sputatrix* [37]. Non-conventional/plesiotypic 3FTx-like homologs in viperids exhibited differences in CRE position and unchanged or reduced expression at 96 hpvm of associated *trans*-factors conserved across expressed 3FTx genes in elapids. There was also an abundance, up to 16, of c/EBP binding sites in the non-toxin group I PLA_2_ homologs in *C. viridis* and *C. tigris.* However, it is difficult to determine from our analysis if a lack of toxin expression is due to differences in predicted CREs and associated *trans-*factors or epigenetic factors (e.g. chromatin organization and gene methylation levels [31, 32]).

Although NFI CREs are present in both elapid and viperid toxin gene promoters, NFI-family TFs appear to likely be of greater importance in the expression of viperid toxin genes in comparison to the elapid *P. textilis* as NFI genes were highly upregulated in both viperids. NFIA, NFIB, and NFIX, are RNA polymerase II core promoter binding TFs with binding sites present and accessible in promoter regions of multiple viperid toxin genes (SVMPs, SVSPs, and group II PLA_2_s) [31, 32], potentially also coordinating the expression of these multiple gene families. NFI-family genes are ubiquitously expressed in different tissues but are known to regulate tissue-specific expression, including mammalian glands [121], through interactions with other TFs, members of the transcription initiation complex, and epigenetic regulators [122, 123]. NFI family binding sites are highly correlated with the center of nucleosome depletion regions, suggesting that their binding directly shapes local chromatin structures and can function as pioneer factors [124, 125]. Pioneer factors are the first factors to engage target sites in chromatin and recruit histone modifying proteins, similar to many other identified viperid toxin gene TFs such as AP-1, CREB3, and FOX family TFs [45, 126]. In addition, we found multiple CREs and associated *trans-*factors that were upregulated in viperids unique to group II PLA_2_s (RAR, USF1, and T3R), as well as varying upregulation of these factors between the two rattlesnake species, highlighting potential regulatory differences that could contribution to venom variation between and within the two snake families.

Post-transcriptional regulation of venom toxins by miRNAs has also been proposed [106]. It was found in Zheng et al., 2023 that most toxin-related families in two elapids (sea snakes *Hydrophis cyanocinctus* and *H. curtus*) were regulated by miRNAs and lncRNAs. We did not find this to be true and identified potential miRNA regulation of only five toxin families (SNAC, PLA_2_, 3FTx, and pseutarin C subunits). We observed that highly expressed miRNAs in the *P. textilis* MVG and UVG did share toxin transcript targets, with additional 3FTxs and pseutarin C non-catalytic subunit transcripts targeted in the UVG. Given that these targeted toxin transcripts are all major components of *P. textilis* venom, and we evaluated only top 10 most abundant miRNAs, there is a high likelihood that miRNAs could be regulating the translation of these toxin transcripts, including the downregulation of toxin translation in UVGs. Post-transcriptional miRNA regulation of venom toxins has been hypothesized to be responsible for ontogenetic venom variation in snakes [106, 127, 128], and our finding that there are miRNAs targeting both pseutarin C subunit transcripts could contribute to the ontogenetic shift in abundance of pseutarin C, as neonate *P. textilis* venoms lack this toxin complex and have venoms that fail to induce clot formation in plasma and whole blood [56, 129]. Venom gland miRNAs from other *P. textilis* age classes will need to be evaluated to test this hypothesis. For *C. viridis*, we only identified two SVMP transcripts targeted, therefore less miRNA regulation of toxin transcripts was seen in the viperid venom gland. Viperid venom gland miRNAs that target SVMP transcripts have also been observed for the Mexican rattlesnakes *C. simus, C. tzabcan* and *C. culminatus* [106, 127], suggesting this could be a common post-transcriptionally regulated toxin gene family in viperids.

Unique miRNA expression signatures were present in the *P. textilis* and *C. viridis* MVGs. This included the presence of *Pte-miR-1* in the *P. textilis* MVG with no known miRNA homology, the absence of *miR-375*, a highly abundant elapid miRNA, from the venom gland of *C. viridis*, and the unique presence of *miR-215-5p* in the *C. viridis* venom gland that was predicted to target SVMP toxin transcripts. Although there was overall a greater number of identified miRNAs in the viperid MVGs, miRNAs in the *P. textilis* MVG were more abundant and target predictions for miRNAs over 100 CPM demonstrated a greater extent of miRNA regulation in comparison to the viperid (Fig. 6). For both elapid and viperid MVGs, miRNAs targeted transcripts for proteins involved in intracellular transport, but a greater extent of metabolic processes were targeted in the elapid MVG, and a greater number of processes related to mRNA processing and response to ER stress were targeted in the viperid MVGs.

As we can now produce snake venom gland organoids [14, 130], it is of even greater relevance to understand the epigenetic and genetic processes that regulate the expression of toxin genes. These insights would be useful to optimize in vitro toxin expression, with applications across fields in biotechnology and therapeutics (e.g., drug development from toxins and antivenom production), and would reduce the need for live venomous snakes to be used in research. The toxin gene regulatory mechanisms we have identified that potentially contribute to venom variation between elapid and viperid snakes will require additional evidence from larger snake venom gland and genome datasets. Our dataset is limited by only having one MVG and UVG for *P. textilis*, but this was done to avoid intraspecific variation in toxin expression, and the logistical and ethical considerations of sacrificing multiple animals. Additional sequencing approaches would be insightful, Assaying for Transposase-Accessible Chromatin followed by sequencing (ATAC-seq) would help to determine which toxin genes are in chromatin accessible regions, and Chromatin Immunoprecipitation followed by sequencing (ChIP-seq) for targeted histone markers (lysine 4 methylation of histone 3 associated with methyltransferases KMT2A, KMT2C, and KMT2D) and TFs (Sp1 and NFI-family TFs) would better determine their relationships to highly expressed elapid toxin genes. Snake-specific TF antibodies are unfortunately not commercially available at this time, but venom gland organoids may offer an alternative approach where CRISPR/Cas9 technology could be used to Epitope Tag endogenous TFs for ChIP-seq (CETCh-seq) [131]. Additionally, all our results are *in silico* and our observations will need experimental validation, especially for determining regions of toxin gene promoters that regulate expression and miRNA targets. Future venom gland organoid experiments could also facilitate such investigations by providing tissue-specific cell cultures for this work.

## Conclusions

Snake venom glands are tractable models to investigate gene regulation, as toxin gene expression and synthesis are upregulated in MVGs, and we can use manual venom milking to experimentally initiate these processes. Elapid and viperid venom glands are thought to share a common evolutionary origin, supported by anatomical and developmental evidence [16], however, these two families have differing venom delivery systems, notably fang and venom gland morphology [21], and major differences in venom composition (Supplemental Table 17). Although there are shared toxin gene families and phylogenetic analysis of toxins suggests venom evolved once at the base of the advanced snake radiation [17], there are distinct toxin genes present in these two snake families and these toxin genes have differing chromosomal locations. Here, we used high-throughput RNA-seq to profile gene expression and miRNAs between MVGs and UVGs in these two snake families, in addition to performing comparative genomic analyses to identify *cis-* and *trans-*acting regulation of venom production in an elapid in comparison to previous viperid datasets (*C. viridis* and *C. tigris*). We identified CREs that are common across multiple toxin genes between these two snake families, but differences in potential key chromatin modifiers, TFs, and miRNAs regulating elapid and viperid toxin expression and synthesis (Supplemental Table 17). Therefore, elapid and viperid venom delivery systems, and their toxin genes and associated regulatory mechanisms, likely evolved independently.

## Methods

### Venom gland collection and RNA-sequencing

An adult *P. textilis* was collected from the Barossa Valley region, South Australia and maintained at Venom Supplies Pty Ltd. (Adelaide, SA, Australia). Venom was milked by placing a pipette tip over the left fang and manual massage of the left venom gland. The right fang and venom gland were left untouched. Four days following venom milking, the snake was humanely euthanized via injection of Sodium Pentobarbital (dose exceeding 100 mg/kg, diluted 5x with WFI and delivered IP), both venom glands dissected (collected under Animal Ethics Approval, project 93/12 issued by SA Pathology/CHN Animal Ethics Committee) and each venom gland separately placed in RNAlater (Thermo Fisher), then shipped to the National University of Singapore. RNA was extracted from the left and right venom glands using a Qiagen RNeasy Mini kit, following the manufacturer’s protocol. For mRNA libraries, 1 µg of total RNA from the MVG and UVG was used as input into Illumina’s TruSeq RNA Sample Preparation v2 kit, and the same done for the small RNA library using Illumina’s TruSeq Small RNA Sample Preparation kit. The small RNA library was loaded onto a 6% PAGE gel (Invitrogen) and a band of ∼170–320 bp was excised from the gel. The size-selected library was then extracted from the PAGE gel and recovered by ethanol precipitation. Quantitation of libraries was performed using Invitrogen’s Picogreen assay and the average library size determined by running the libraries on a Bioanalyzer DNA 1000 chip (Agilent). Library concentration was normalized to 2 nM and concentrations, and validated by qPCR on a ViiA-7 real-time thermocycler (Applied Biosystems) using qPCR primers recommended in Illumina’s qPCR protocol and Illumina’s PhiX control library used as a standard. Libraries were then pooled at equal volumes and the two library types sequenced on separate lanes of an Illumina HiSeq2500 rapid run at a final concentration of 11 pM, a read-length of 101 bp paired-end for the mRNA library and 51 bp single-end for the small RNA library.

An adult *C. viridis* rattlesnake of the same northeastern Colorado locality as used by Schield et al. (2019) for prior *C. viridis* genome sequencing was collected and venom milked. Four days following venom milking, the snake was humanely euthanized and both venom glands dissected (IACUC protocol no. 9204, granted by the University of Northern Colorado). RNA isolation was completed for both venom glands combined, following the TRIzol reagent (Thermo Fisher Scientific) manufacturer’s protocol, with an additional overnight − 20 °C incubation in 300 µL 100% ethanol with 40 µL 3 M sodium acetate, centrifugation and supernatant removal following the incubation, and total RNA resuspended in nuclease-free H_2_O. mRNA library preparation was completed using the same protocol as described above for *P. textilis* with paired-end sequencing to a read length of 150 bp. The small RNA library preparation was completed with 1 µg of total RNA isolated with a mirVana RNA isolation kit (Thermo Fisher Scientific), and following the NEBNext Small RNA Library Prep Set for Illumina (New England BioLabs) manufacture’s protocol. AMPure XP Beads (Beckman Coulter) were used for size selection (110–160 bp). These small RNA libraries were sequenced to 75 bp, single-end.

### Venom gland *de novo* transcriptome assembly and toxin annotation

Sequenced reads were assessed with the Java program FastQC (Babraham Institute Bioinformatics, UK) to confirm that all adapters and low quality reads (< Q20) were removed before assembly. Low quality reads were trimmed and adaptors removed using Trimmomatic (79) with a sliding window of 4 nucleotides and a threshold of phred 30. To obtain a comprehensive *de novo* venom gland transcriptome assembly for *P. textilis*, separate assemblies for each gland were completed, and three assemblers used: (1) ABySS (release v1.5.0) [132, 133] with paired-end default parameters and k-mer sizes 30 to 66, increased in increments of 4, and merged with TransABySS (v1.5.1) [134], (2) Trinity (release v2014-07-17) [135] with genome-guided assembly default parameters using Bowtie2 (v2.2.6) [136] aligned reads to the *P. textilis* genome (assembly EBS10Xv2-PRI), and (3) Extender [137] with 10,000 starting seeds, where seeds were reads first merged with PEAR (Paired-End read mergeR; v0.9.6 using default parameters) [138] and seed extensions required 100 nucleotide overlaps and quality scores of at least 30. For *C. viridis*, a *de novo* venom gland transcriptome assembly was completed using the same approached detailed above for *P. textilis*, except excluding ABySS and instead a Trinity assembly using *de novo* parameters, based still on a de Bruijn graph algorithm. For the *de novo* assembled transcriptomes for both species, contigs less than 150 nucleotides and redundancies between assemblies were removed with CD-HIT (v4.6.6) [139, 140] and exonerate (fastanrdb) [141]. For *P. textilis*, coding contigs were then identified with EvidentialGene (downloaded May 2018) [142]. Abundances of the *P. textilis* coding contig set and all assembled *C. viridis* contigs were determined with RSEM (RNA-seq by Expectation Maximization, v1.3.0) [143], using the aligner Bowtie2 (v2.2.6) [136]. Contigs less than 1 TPM (Transcript Per Million) were filtered out, and the remaining contigs annotated with Diamond [144] and BLASTx (E-value 10^− 05^ cut-off) searches against the *P. textilis* genome-predicted protein set and the National Center for Biotechnology Information (NCBI) non-redundant protein database. Only complete *de novo* assembled myotoxin transcripts, identified using a ‘myotoxin’ search term, were used for the remaining analysis for *C. viridis*, but OrfPredictor (v3) [145] was used to identify all coding and protein sequence prediction with BLASTx input to aid in proper transcript translation for *P. textilis*. Venomix [146] was used to help to identify all toxin transcripts in addition to toxins being manually evaluated to determine if venom proteins were full-length, shared sequence identity to currently known toxins, and contained a conserved signal peptide sequence within each venom protein family for *P. textilis*.

### Venom gland transcript expression, annotation, and *cis-*regulatory element predictions

From *P. textilis* genome annotations (assembly EBS10Xv2-PRI), the global transcriptome (34,614 predicted transcripts), plus the final *de novo* assembled and annotated toxin transcript set, was used as a reference for aligning reads originating from the *P. textilis* MVG and UVG. For rattlesnake MVGs and UVGs, the following NCBI data sets were used: SRR11524062 (*C. tigris* UVG), SRR11524063 (*C. tigris* UVG), SRR11524059 (*C. tigris* 24 hpvm), SRR11524060 (*C. tigris* 24 hpvm), SRR11524050 (*C. tigris* 96 hpvm), SRR11524051 (*C. tigris* 96 hpvm), SRR7401989 (*C. viridis* UVG), SRR7402004 (*C. viridis* 24 hpvm), and SRR7402005 (*C. viridis* 72 hpvm). Reads from these data sets were aligned to annotated transcriptomes from genome assemblies (UTA_CroVir_3.0 and ASM1654583v1 for *C. viridis* and *C. tigris*, respectively, and the *C. viridis* global transcriptome provided by Blair Perry [45]), in addition to myotoxin transcripts from the *de novo* assembled venom gland transcriptome for *C. viridis*. Toxin transcripts were identified from transcriptomes using key word searches for each venom protein that had been annotated as toxin genes (5’nucleotidase, bradykinin-potentiating peptide, cysteine-rich secretory protein, L-amino acid oxidases, C-type lectin, phospholipase A_2_, snake venom metalloproteinase, snake venom serine protease, and vascular endothelial growth factor) in Viperid genomes (Supplemental Tables 5 and 6 for *C. viridis* and *C. tigris*, respectively) [31, 32]. Transcript abundances were determined with Bowtie2 (v2.2.6) [136] read alignments and RSEM [143], this approach was used to maintain consistency between transcript quantifications from both *de novo* assembled and genome-referenced transcriptomes. RSEM output of expected counts, transcript length and FPKM were used as input into GFOLD [61] to identify transcript fold-changes between the conditions. A Gene Set Enrichment Analysis (GSEA) [71, 72] was performed using RSEM estimated transcript abundances as input, the set parameters were the following: log2 ratio of classes with 10,000 permutations. Transcripts were searched against the UniProt *Homo sapiens* protein database [103] with Diamond BLASTx (v0.8.34) [144] to identify orthologs. UniProt accessions were then entered into DAVID Bioinformatics Resources 6.8 [147] to identify functional annotations and pathways. In addition, gene networks were constructed using the ClueGo app plug-in [75] in Cytoscape [76]. *Cis*-regulatory element predictions were completed upstream toxin gene transcription start sites, up to 700 bp if available, either from published literature or from tBLASTn searches against the genomes of *P. textilis* (assembly EBS10Xv2-PRI), *C. viridis* (UTA_CroVir_3.0), or *C. tigris* (ASM1654583v1) using translated full-length transcript sequences for toxins. Promoter sequences were then entered into the online server AliBaba2.1 (http://gene-regulation.com/pub/programs/alibaba2/) with the TRANSFAC 4.0 database embedded within the webserver. GFOLD determined fold-changes in MVGs for *trans*-factors associated with CREs were then evaluated.

### Venom gland microRNA expression and target prediction

TrueSeq small RNA library adapters were trimmed with the fastx_clipper tool, provided in the FASTX-Toolkit (Hannon lab Cold Spring Harbor Laboratory). Trimmomatic [148] was used to filter out low quality (< Q20) reads, evaluated with a sliding window of 4 nucleotides. To filter out rRNA, reads were then aligned with Bowtie2 (v2.2.6) to known snake rRNA sequences. Non-rRNA reads were used as input into miRDeep2 (v2.0.1.2) [149] to identify species-specific miRNAs from the *P. textilis* genome (GCF900518735.1, assembly EBS10Xv2-PRI) and *C. viridis* (UTA_CroVir_3.0). miRNAs were identified by lengths of 18–23 bp, alignment to genomes, and the presence of a transcribed hairpin structure, predicted by miRDeep2 [149]. Expression levels of miRNAs in venom glands were estimated by normalization to Counts Per Million (CPMs; CPM = mature miRNA reads / total mapped miRNA reads * 10^6^). Toxin transcript target prediction was performed with the miRanda position-weighted local alignment algorithm using a criteria of -19 kcal/mol or less free energy pairing between miRNA:mRNA [150, 151], as used previously to identify toxin transcripts targeted by venom gland miRNAs [106, 128]. Contigs coding for full length venom proteins from the *de novo* assembled transcriptome from *P. textilis* and toxin transcripts from the annotated in *C. viridis* genome, in addition to the *de novo* assembled myotoxin transcript, were used as input. A stricter free energy value of at least − 30 kcal/mol was used for target identification from genome annotated transcriptome datasets to reduce false positives and only transcripts evaluated that were co-expressed (at least over 10 TPM) in the venom glands. Non-toxin transcripts targeted were searched against the UniProt *Homo sapiens* protein database [103] with Diamond BLASTx (v0.8.34) [144] to identify orthologs, and accessions were then entered into DAVID Bioinformatics Resources 6.8 [147] to identify functional annotations and pathways. In addition, gene networks were constructed using the ClueGo app plug-in [75] in Cytoscape [76]. All commands and scripts/software links used are available on https://github.com/CModahl/ElapidvsViperid.git.

### Electronic supplementary material

Below is the link to the electronic supplementary material.

Supplementary Material 1

Supplementary Material 2

Supplementary Material 3

Supplementary Material 4

Supplementary Material 5

Supplementary Material 6

Supplementary Material 7

Supplementary Material 8

Supplementary Material 9

## Data Availability

The datasets supporting the conclusions of this article are available in the National Center for Biotechnology Information (NCBI) repository, under BioProject ID PRJNA931953 (BioSample SAMN33139130 for *P. textilis* and SAMN33139131 for *C. viridis;* SRR23348481-SRR23348476). All commands, scripts/software links, and a fasta file of all promoter sequences are available on https://github.com/CModahl/ElapidvsViperid.git. Toxin contigs from the *de novo* assembly of the *P. textilis* venom gland transcriptome have been submitted to GenBank under accession numbers PP227363-PP227414 with CDS regions annotated.
