# Supplementary material for: Distinct regulatory networks control toxin gene expression in elapid and viperid snakes
Source: BMC Genomics. 2024 Feb 16;25:186. doi: 10.1186/s12864-024-10090-y (PMC10874052; doi:10.1186/s12864-024-10090-y)
Supplement: Supplementary file 9 — Supplementary Material 9 [file 12864_2024_10090_MOESM9_ESM.pdf]

Supplemental Figure 1

A

Milked venom gland

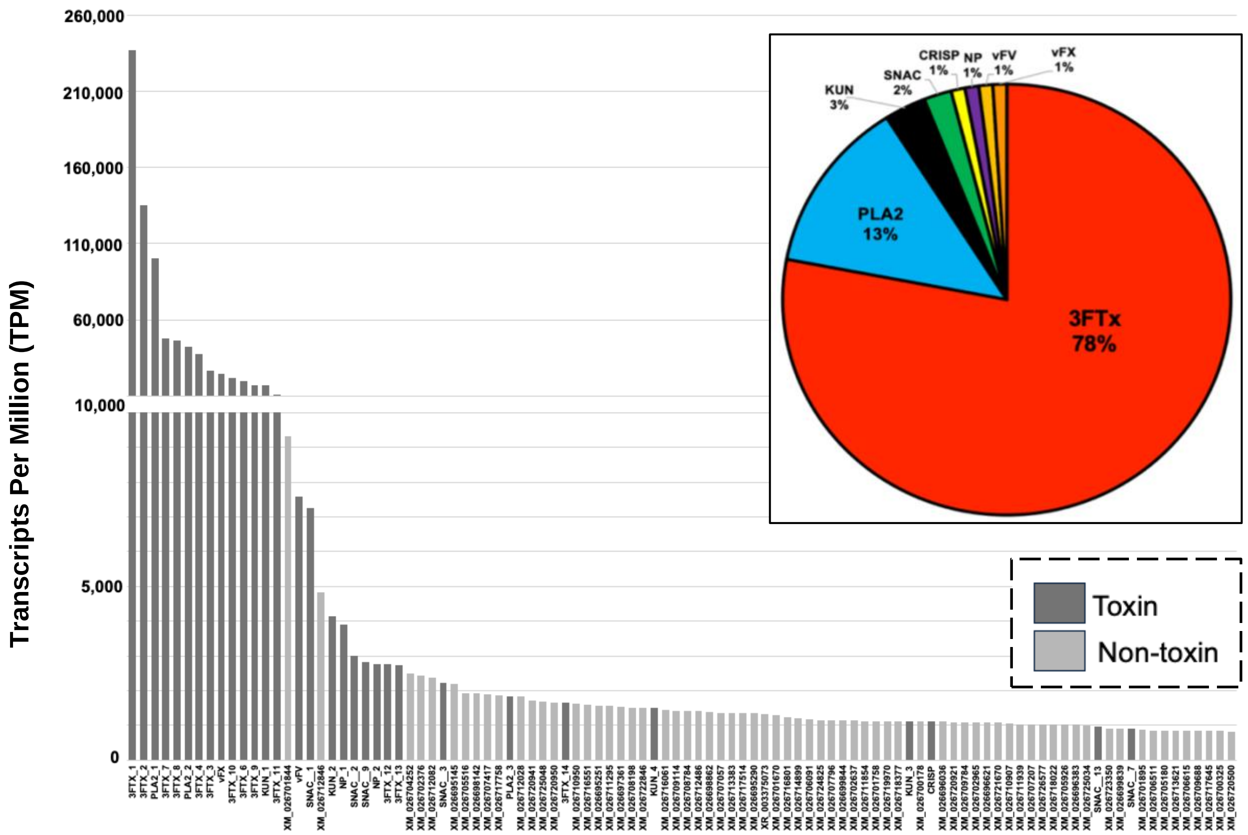

B

Unmilked venom gland

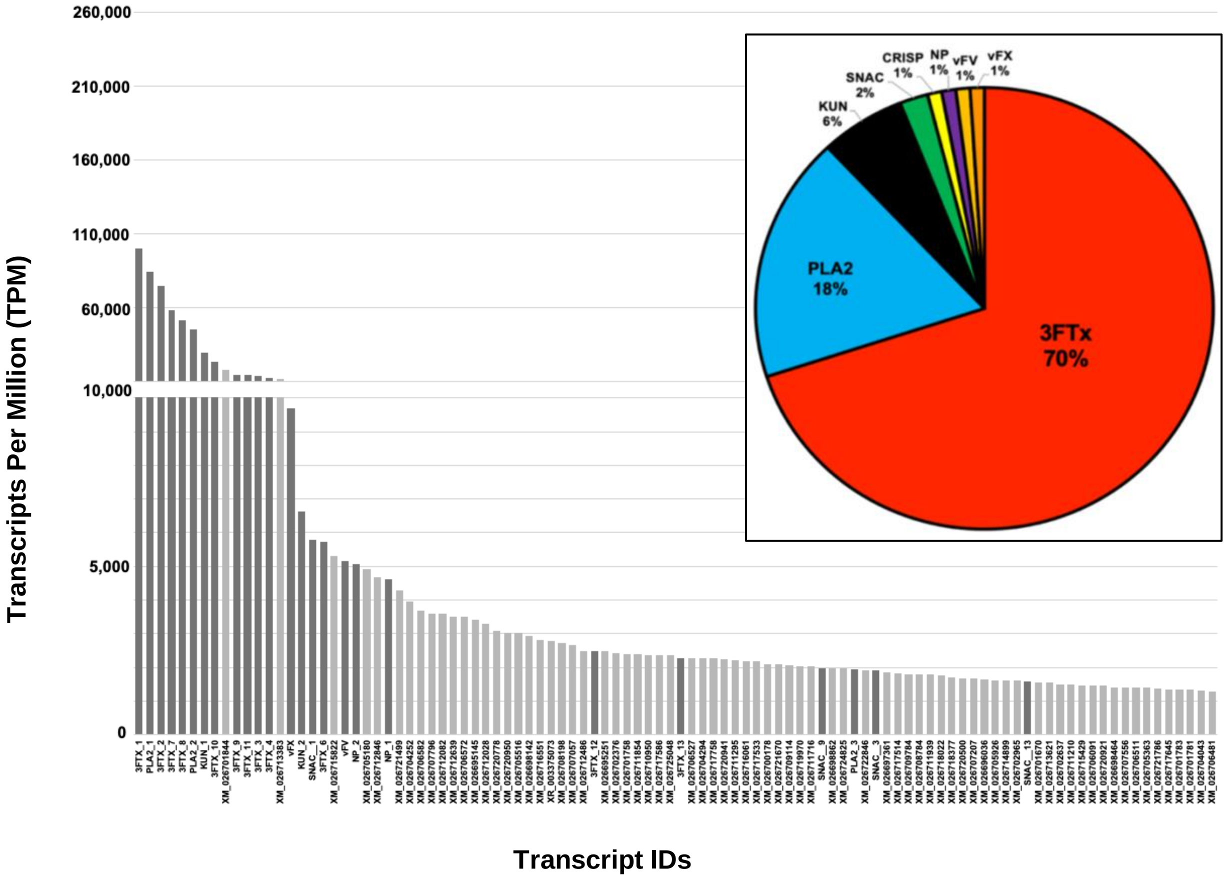

Supplemental Figure 1. Toxin and non-toxin expression in a *Pseudonaja textilis* venom gland 96 hours post milking in comparison to an unmilked venom gland. Toxin transcripts (dark grey bars with transcript identities determine from *de novo* assembled venom gland transcriptomes) are expressed in high abundance (TPM; transcripts per million) in comparison to non-toxin transcripts (light grey bars with accession numbers from *P. textilis* genome annotations) in the top 100 expressed transcripts in (A) milked and (B) unmilked venom glands. Pie charts (insets in respective panels) show the percentages of total toxin reads belonging to each toxin superfamily, with toxin superfamilies that made up less than 1% excluded due to low abundance. Toxin identifications are as follows: 3FTx = three-finger toxin, CRISP = cysteine-rich secretory protein; KUN = Kunitz-type serine proteinase inhibitor; NP = natriuretic peptide; PLA2 = phospholipase A_2; SNAC = snake venom C-type lectin; vFA = venom factor V (pseutarin C non-catalytic subunit); vFX = venom factor X (pseutarin C catalytic subunit).

Supplemental Figure 2

A Three-finger toxins

Long-chain like

```
3FTx_1                        LICYLDFS-VPHTCAPGEKLCYTRTWNDG----RGTRIERGCAATCPIPKKPEIHVTCCSTDRCNPHPKEKPH
Pseudonaja_LC                 LICYLDFS-VPHTCAPGEKLCYTRTWNDG----RGTRIERGCAATCPIPKKPEIHVTCCSTDRCNPHPKQKPH
```

Long-chain

```
Notechis                      LICYMGPK-TPRTCPRGQNLCYTKTWCDAFCSSRGKVVELGCAATCPIA-KSYEDVTCCSTDNCNPFPVRPRPHP-------------
Oxyuranus                     RRCFITPDVRSERCPPGQEVCYTKTWCDGFCGSRGKRVDLGCAATCPTPKKKGIDIICCSKDNCNTFPKWP-----------------
3FTx_3                        RTCFITPDVKSKPCPPGEEVCYTKTWCDGFCGIRGKRVDLGCAATCPTPKKTGIDIICCSTDDCNTFPLRP------RGRLSSIKDHP
3FTx_6*                       RTCFITPDVKSKPCPPGQEVCYTKTWCDGFCGIRGKRVDLGCAATCPTPKKTGIDIICCSTDDCNTFPLRP------RGRLSSIKDHP
Pseudonajatoxinb_homolog*     RTCFITPDVKSKPCPPGQEVCYTKTWCDGFCGIRGKRVDLGCAATCPTPKKTGIDIICCSTDDCNTFPLRP------RGRLSSIKDHP
3FTx_5                        RTCFITPDVKSKPCPPGQEVCYTETWCDGVCGIRGKRVDLGCAATCPTPKKTGIDIICCSTDDCNTFPLRP------RGRLSSIKDHP
3FTx_2                        RTCFITPDVKSKPCPPGQEVCYTETWCDGFCGIRGKRVELGCAATCPTPKKTGIDIICCSTDDCNTFPLRP------RGRLSSIKDHP
Pseudonajatoxinb              RTCFITPDVKSKPCPPGQEVCYTETWCDGFCGIRGKRVELGCAATCPTPKKTGIDIQCCSTDDCNTFPLRP-----------------
Drysdalia                     RKCYKTHPYKSEPCAPGENLCYTKTWCDFRCSQLGKAVELGCAATCPTT-KPYEEVTCCSTDDCNRFPNWERPRPRPRGLLSSIMDHP
Austrelaps                    FSCYKTPDVKSEPCAPGENLCYTKTWCDRFCSIRGKVIELGCAATCPPA-EPRKDITCCSTDNCNPHPAH------------------
3FTx_4                        RTCFKTPYVKSEPCPPGQEVCYTKTWCDRFCSIRGRVIELGCAATCPPA-GPKEDVTCCSTDNCNTHP--------------------
Demansia                      RTCLKTPEVKSEPCPPGQEVCYTKAWCDRMCSFRGKVIELGCAATCPRQ-EPGKEITCCSTDDCNTHP--------------------
```

Short-chain

```
3FTx_9                        LICHDSENLDDHVVCKEDETMCYQYTFVPFRDFEVVARGCSP-SCPEEEDAVCCSTDLCNK
Oxyuranus                     LTC--YMNPSGTMVCKEHETMCYRLIVWTFQYHVLYLKGCSS-SCPGGNNCACCSTDLCNN
3FTx_13                       LTC--YNTLGGTVVCKPHETICYEHTFCPFPNRVIFLRGCST-SCPGGNNPVCCSTDLCNL
3FTx_14                       LTC--YKGYHDTVVCKPHETICYEHTFCPFPNRVIFLRGCST-SCPGGNNPVCCSTDLCNL
P.textilis_SC7                LTC--YKRYFDTVVCKPQETICYRYIIPATHGNAITYRGCST-SCPSGIRLVCCSTDLCNK
3FTx_12                       LTC--NKSYYDTVVCKPHETICYRYHVPATHGNVITVRGCST-SCPGGNNPVCCSTDLCNL
P.textilis_SC6                LTC--YKSLSGTVVCKPHETICYRRLIPATHGNAIIDRGCST-SCPGGNRPVCCSTDLCNK
3FTx_11                       LTC--YKGYHDTVVCKPHETICYRYALPATHGNAVTLRGCGT-SCPEGIRPVCCSTDLCNK
P.textilis_SC8                LTC--YKGYHDTVVCKPHETICYEYFIPATHGNVITTRGCST-SCPGGIRPVCCSTDLCNN
3FTx_7**                      LTC--YKGYHDTVVCKPHETICYEYFIPATHGNAILARGCGT-SCPGGIRPVCCRTDLCNK
P.textilis_SC1/5**            LTC--YKGYHDTVVCKPHETICYEYFIPATHGNAILARGCGT-SCPGGIRPVCCRTDLCNK
P.textilis_SC4                LTC--YKGYHDTVVCKPHETICYEYFIPATHGNAILARGCGT-SCPGGIRPVCCRTDLSNK
P.textilis_SC3                LTC--YKGYHDTVVCKPHETICYRYLVPATHGNAIPARGCGT-SCPGGNHPVCCSTDLCNK
3FTx_8***                     LTC--YKGYHDTVVCKPHETICYRYLIPATHGNAIPARGCGT-SCPGGNHPVCCSTDLCNK
P.textilis_SC2***             LTC--YKGYHDTVVCKPHETICYRYLIPATHGNAIPARGCGT-SCPGGNHPVCCSTDLCNK
3FTx_10                       LTC--YKGYHDTVVCKPHETICYRYLIPATHGNAILARGCGT-SCPGGNHPVCCSTDLCNK
```

B Cysteine-rich secretory proteins

```
Pseudechis_australis          TADFASESSNKKNYQKEIVDKHNALRRSVKPTARNMLQMKWNSRAAQNAKRWANRCTFAHSPPNKRTVGKLRCGENIFMSSQPFPWSGVV
Pseudechis                    TVDFASESSNKKNYQKEIVDKHNALRRSVKPTARNMLQMKWNSHAAQNAKRWADRCTFAHSPPNTRTVGKLRCGENIFMSSQPFPWSGVV
CRISP_1*                      TVDFASESSNKNDYQKEIVDKHNDLRRSVKPTARNMLQMKWNSRAAQNAKRWANRCTFAHSPPYTRTVGKLRCGENIFMSSQPFAWSGVV
Pseudonaja_textilis*          TVDFASESSNKNDYQKEIVDKHNDLRRSVKPTARNMLQMKWNSRAAQNAKRWANRCTFAHSPPYTRTVGKLRCGENIFMSSQPFAWSGVV
Oxyuranus                     TVDFASESSNKKDYRKEIVDKHNDLRRSVKPTARNMLQMKWNSRAAQNAKRWANRCTFAHSPPYTRTVGKLRCGENIFMSSQPFAWSGVV
Drysdalia                     TVDFASESSNKKDYRKEIVDKHNALRRSVKPTARNMLQMEWNSHAAQNAKRWADRCTFAHSPPHTRTVGQLRCGENIFMSSQPFAWSGVV
Notechis                      TVDFASESSNKKDYQKEIVDKHNALRRSVKPTARNMLRMEWNSHAAQNAKRWADRCTFAHSPPHTRTVGKLRCGENIFMSSQPFAWSGVV
Austrelaps                    TVDFASESSNKKDYRKEIVDKHNALRRSVKPTARNMLRMEWNSRAAQNAKRWADRCTFAHSPPHTRTVGKLRCGENIFMSTQPFAWSGVV
```

```
Pseudechis_australis     QAWYDEIKNFVYGIGAKPPGSVIGHYTQVVWYKSYLIGCASAKCSSSKYLYVCQYCPAGNIRGSIATPYKSGPPCADCPSACVNKLCTNP
Pseudechis               QAWYDEIKNFVYGIGAKPPGSVIGHYTQVVWYKSHLIGCASAKCSSSKYLYVCQYCPAGNIRGSIATPYKSGPPCADCPSACVNRLCTNP
CRISP_1*                 QAWYDEVKKFVYGIGAKPPSSVTGHYTQVVWYKSHLLGCASAKCSSTKYLYVCQYCPAGNIVGSIATPYKSGPPCGDCPSACDNGLCTNP
Pseudonaja_textilis*     QAWYDEVKKFVYGIGAKPPSSVTGHYTQVVWYKSHLLGCASAKCSSTKYLYVCQYCPAGNIVGSIATPYKSGPPCGDCPSACDNGLCTNP
Oxyuranus                QAWYDEVKKFVYGIGAKPPSSVIGHYTQVVWYKSHLLGCASAKCSSTKYLYVCQYCPAGNIIGSIATPYKSGPPCGDCPSACDNGLCTNP
Drysdalia                QAWYDEVKKFVYGIGAKPPGSVIGHYTQVVWYKSHLLGCASAKCSSTKYLYVCQYCPAGNIRGSIATPYKSGPTCGDCPSACVNGLCTNP
Notechis                 QAWYDEVKKFVYGIGAKPPGSVIGHYTQVVWYKSHLLGCASAKCSSTKYLYVCQYCPAGNIRGSIATPYKSGPTCGDCPSACVNGLCTNP
Austrelaps               QAWYDEVKKFVYGIGAKPPGSVIGHYTQVVWYKSHLLGCASAKCSSTKYLYVCQYCPAGNIRGSIATPYKSGPACGDCPSACVNGLCTNP

Pseudechis_australis     CKRNNDFSNCKSLAKKSKCQTEWIKKKCPASCFCHNKII
Pseudechis               CNYNNDFSNCKSLAKKSKCQTEWIKKKCPASCFCHNKII
CRISP_1*                 CKHNDDLSNCKTLVKKHKCQTEWIKSKCPATCFCRTEII
Pseudonaja_textilis*     CKHNDDLSNCKTLVKKHKCQTEWIKSKCPATCFCRTEII
Oxyuranus                CKHNDDLSNCKPLAKKSKCQTEWIKSKCPATCFCRTEII
Drysdalia                CKYEDAFTNCNELAKETKCKTEWIKSKCPATCFCHTEII
Notechis                 CKYEDDFSNCKALAKNSKCQTEWIKSKCPAACFCHNKII
Austrelaps               CKYEDAFTNCKALAKKTKCKTEWIKSKCPATCFCHNKII
```

C Kunitz-type serine protease inhibitors

```
KUN_3                    KDRPKFCELPADIGPCDDFTGAFHYSPREHECIEFIYGGCKGNANNFNTQEECESACAA-
Textilinin-6             KDRPKFCELPADIGPCDDFTGAFHYSPREHECIEFIYGGCKGNANNFNTQEECESTCAA-
Scutellin-3              KDRPKFCELPADIGPCEDFTGAFHYSPREHECIEFIYGGCKGNANNFNTLEECESACAA-
Microlepidin-3           KDRPKFCELPADIGPCEDFTGAFHYSPREHECIEFIYGGCEGNANNFNTLEECESACAA-
KUN_4                    KDRPKFCELLPDTGPCDDFTGAFHYSTRDRECIEFIYGGCGGNANKFNTLEECESTCARK
Textilinin-5             KDRPKFCELLPDTGSCEDFTGAFHYSTRDRECIEFIYGGCGGNANNFITKEECESTCAA-
Textilinin-7             KDRPKFCELLPDTGSCEDFTGAFHYSTRDRECIEFIYGGCGGNANNFKTLEECESTCAA-
Textilinin-2             KDRPELCELPPDTGPCRVRFPSFYYNPDEQKCLEFIYGGCEGNANNFITKEECESTCAA-
KUN_1*                   KDRPDFCELPADTGPCRVRFPSFYYNPDEKKCLEFIYGGCEGNANNFITKEECESTCAA-
Textilinin-1*            KDRPDFCELPADTGPCRVRFPSFYYNPDEKKCLEFIYGGCEGNANNFITKEECESTCAA-
Mulgin-3                 KDRPDFCELPADTGPCRVGFPSFYYNPDEKKCLEFIYGGCQGNANNFITKEECESTCAA-
Textilinin-3             KDRPNFCKLPAETGRCNAKIPRFYYNPRQHQCIEFLYGGCGGNANNFKTIKECESTCAA-
KUN_2                    KDRPEFCELPADTGSCKGNVPRFYYNADHHQCLKFIYGGCGGNANNFKTIEECKSTCAA-
Textilinin-4             KDHPKFCELPADTGSCKGNVPRFYYNADHHQCLKFIYGGCGGNANNFKTIEECKSTCAA-
```

D Group I phospholipase A_2

```
textilotoxin_C           ARIPPLPLNLIQFSNMIKCTIPGSQPLLDYANYGCYCGPGNNGTPVDDVDRCCQAHDECYDEASNHG-CY-----PELTLYDYYCDTGV
textilotoxin_A           SDIPPLPLNLVQFSYLIRCANKYKRPGWHYANYGCYCGSGGRGTPVDDVDRCCQAHDKCYEDAEKLG-CY-----PKWTTYYYYCGANG
textilotoxin_B           --------DLVEFGFMIRCANRNSQPAWQYMDYGCYCGKRGSGTPVDDVDRCCQTHNECYDEAAKIPGCK-----PKWTFYFYQCGSGS
textilotoxin_D           -SIPRPSLNIMLFGNMIQCTIPCEQSWLGYLDYGCYCGSGSSGIPVDDVDKCCKTHDECYYKAGQIPGCSVQPNEVFNVDYSYECNEG-
Austrelaps               SNIPLPSLDFEQFGKMIQCTIPCEESCLAYMDYGCYCGPGGSGTPSDELDRCCQTHDNCYAEAGKLPACKAMLSEPYNDTYSYSCIER-
Tropidechis              --IPARPLNLYQFGNMIQCANHGRRPTWHYMDYGCYCGKGGSGTPVDELDRCCQIHDDCYGEAEKLPACNYMMSGPYYNTYSYECNEG-
Notechis                 --------NLYQFGNMIQCANHGRRPTRHYMDYGCYCGKGGSGTPVDELDRCCQTHDDCYGEAEKLPACNYMMSGPYYNTYSYECNEG-
Oxyuranus                ARIPPLPLSLLNFANLIECANHGTRSALAYADYGCYCGKGGRGTPLDDLDRCCHVHDDCYGEAEKLPACNYLMSSPYFNSYSYKCNEG-
PLA2_1*                  -RIPPLPLSLDDFSNLITCANRGSRSWLDYAHYGCFCGSGGSGTPVDDLDRCCQVHDNCFGDAEKLPACNYLFSGPYWNPYSYKCNEG-
Pseudonaja_1*            -RIPPLPLSLDDFSNLITCANRGSRSWLDYAHYGCFCGSGGSGTPVDDLDRCCQVHDNCFGDAEKLPACNYLFSGPYWNPYSYKCNEG-
PLA2_3                   -RIPPLPLSLVEFRILIKCANHNSRNVLDYADYGCYCGKGGSGTPVDELDRCCQAHDYCYDDAEKLPACNYLFSGPYWNPYSYKCNEG-
PLA2_2                   -RIPPLPLSLVEFRILIKCANHNSRNVLDYADYGCYCGKGGSGTPVDELDRCCQAHDYCYDDAEKLPACNYRFSGPYWNPYSYKCNEG-
Pseudonaja_2             -RIPPLPLSLVEFRILIKCANHNSRNVLDYADYGCYCGKGGSGTPVDELDRCCQAHDYCYDDAEKLPACNYRFSGPYWNPYSYKCNEG-

textilotoxin_C           -PYC-KARTQCQVFVCGCDLAVAKCLAGATYNDENKNINTGER--CQ
textilotoxin_A           -PYC-KTRTKCQRFVCNCDVVAADCFASYPYNRRYWFYSNKKR--CR
textilotoxin_B           QFTCRKSKDVCRNVVCDCDFKAALCLTGARYNSANYNIDIKTH--CR
textilotoxin_D           QLTCNESNNECEMAVCNCDRAAAICFARFPYNKNYWSINTEIH--CR
Austrelaps               QLTCNDDNDECKAFICNCDRAAVICFSGAPYNDSNYDIGTIEH--CK
Tropidechis              ELTCKDNNDECKAFICNCDRTAAICFARTPYNDANWNIDTKTR--C-
Notechis                 ELTCKDNNDECKAFICNCDRTAAICFARAPYNDANWNIDTKTR--CQ
Oxyuranus                KVTCTDDNDECKAFICNCDRTAAICFAGATYNDENFMISKKRNDICQ
PLA2_1*                  EITCTDDNDECAAFICNCDRTAAICFAGATYNDENFMVTIKKKNICQ
Pseudonaja_1*            EITCTDDNDECAAFICNCDRTAAICFAGATYNDENFMVTIKKKNICQ
PLA2_3                   EITCTDDNDECAAFICNCDRTAAICFAGATYNDENFMVTIKKKNICQ
PLA2_2                   EVTCTDDNDECKAFICNCDRTAAICFAGAPYNDENFMITTKKKNICQ
Pseudonaja_2             EVTCTDDNDECKAFICNCDRTAAICFAGAPYNDENFMITIKKKNICQ
```

E Coagulation factor V

```
FV                       AQLREYHIAAQLEDWDYNPQPEELSRLSESDLTFKKIVYREYELDFKQEKPRDELSGLLGPTLRGEVGDILIIYFKNFATQPVSIHPQSAVY
vFV                      AQLREYHIAAQLEDWDYNPQPEELSRLSESDLTFKKIVYREYELDFKQEKPRDALSGLLGPTLRGEVGDSLIIYFKNFATQPVSIHPQSAVY
Pseutarin C              AQLREYHIAAQLEDWDYNPQPEELSRLSESDLTFKKIVYREYELDFKQEEPRDALSGLLGPTLRGEVGDSLIIYFKNFATQPVSIHPQSAVY
Omicarin C               AQLREYRIAAQLEDWDYNPQPEELSRLSESELTFKKIVYREYELDFKQEKPRDELSGLLGPTLRGEVGDILIIYFKNFATQPVSIHPQSAVY
Oscutarin C              AQLREYRLAAQLEDWDYNPQPEELSRLSESDLTFKKIVYREYELDFKQEKPRDELSGLLGPTLRGEVGDSLIIYFKNFATQPVSIHPQSAVY

FV                       NKWSEGSSYSDGTSDVERLDDAVPPGQSFKYVWNITAEIGPKKADPPCLTYAYYSHVNMVRDFNSGLIGALLICKEGSLNANGSQKFFNREY
vFV                      NKWSEGSSYSDGTSDVERLDDAVPPGQSFKYVWNITAEIGPKKADPPCLTYAYYSHVNMVRDFNSGLIGALLICKEGSLNANGSQKFFNREY
Pseutarin C              NKWSEGSSYSDGTSDVERLDDAVPPGQSFKYVWNITAEIGPKKADPPCLTYAYYSHVNMVRDFNSGLIGALLICKEGSLNANGSQKFFNREY
Omicarin C               NKWSEGSSYSDGTSDVERLDDAVPPGQSFKYVWNITAEIGPKKADPPCLTYAYYSHVNMVRDFNSGLIGALLICKEGSLNANGAQKFFNREY
Oscutarin C              NKWSEGSSYSDGTSDVERLDDAVPPGQSFKYVWNITAEIGPKKADPPCLTYAYYSHVNMVRDFNSGLIGALLICKEGSLNADGAQKFFNREY
```

```
FV              VLMFSVFDESKNWYRKPSLQYTINGFANGTLPDVQACAYDHISWHLIGMSSSPEIFSVHFNGQTLEQNHYKVSTINLVGGASVTANMSVSRT
vFV             VLMFSVFDESKNWYRKPSLQYTINGFANGTLPDVQACAYDHISWHLIGMSSSPEIFSVHFNGQTLEQNHYKVSTINLVGGASVTADMSVSRT
Pseutarin C     VLMFSVFDESKNWYRKPSLQYTINGFANGTLPDVQACAYDHISWHLIGMSSSPEIFSVHFNGQTLEQNHYKVSTINLVGGASVTADMSVSRT
Omicarin C      VLMFSVFDESKNWYRKPSLQYTINGFANGTLPDVQACAYDHISWHLIGMSSSPEIFSVHFNGQTLEQNHYKVSTINLVGGASVTANMSVSRT
Oscutarin C     VLMFSVFDESKNWYRKPSLQYTINGFANGTLPDVQACAYDHISWHLIGMSSSPEIFSVHFNGQTLEQNHYKVSTINLVGGASVTANMSVSRT

FV              GKWLISSLVAKHLQAGMYGYLNIKDCGNPDTLTRKLSFRELRRIMNWEYFIAAEEITWDYAPEIPSSVDRRYKAQYLDNFSNFIGKKYKKAV
vFV             GKWLISSLVAKHLQAGMYGYLNIKDCGNPDTLTRKLSFRELMKIKNWEYFIAAEEITWDYAPEIPSSVDRRYKAQYLDNFSNFIGKKYKKAV
Pseutarin C     GKWLISSLVAKHLQAGMYGYLNIKDCGNPDTLTRKLSFRELMKIKNWEYFIAAEEITWDYAPEIPSSVDRRYKAQYLDNFSNFIGKKYKKAV
Omicarin C      GKWLISSLVAKHLQAGMYGYLNIKDCGHPNTLTRKLSFRELRRIMNWEYFIAAEEITWDYAPEIPSSVDRRYKAQYLDNFSNFIGKKYKKAV
Oscutarin C     GKWLISSLVAKHLQAGMYGYLNIKDCGNPDTLTRKLSFRERRRIMKWEYFIAAEEITWDYAPEIPSSVDRRYKAQYLDNFSNFIGKKYKKAV

FV              FRQYKDSNFTKPTYAIWPKERGILGPVIRAKVRDTISIVFKNLASRPYSIYVHGVSVSKDAEGAIYPSDPKENITHGKAVEPGQVYTYKWTV
vFV             FRQYEDGNFTKPTYAIWPKERGILGPVIKAKVRDTVTIVFKNLASRPYSIYVHGVSVSKDAEGAIYPSDPKENITHGKAVEPGQVYTYKWTV
Pseutarin C     FRQYEDGNFTKPTYAIWPKERGILGPVIKAKVRDTVTIVFKNLASRPYSIYVHGVSVSKDAEGAIYPSDPKENITHGKAVEPGQVYTYKWTV
Omicarin C      FRQYEDGNFTKPTYAIWPKERGILGPVIKAKVRDTVTIVFKNLASRPYSIYVHGVSVSKDAEGAIYPSDPKENITHGKAVEPGQVYTYKWTV
Oscutarin C     FRQYEDSNFTKPTYAIWPKERGILGPVIKAKVRDTVTIVFKNLASRPYSIYVHGVSVSKDAEGAVYPSDPKENITHGKAVEPGQVYTYKWTV

FV              LDTDEPTVKDSECITKLYHSAVDMTRDIASGLIGPLLVCKHKALSVKGVQNKADVEQHAVFAVFDENKSWYLEDNIKKYCSNPSTVKKDDPK
vFV             LDTDEPTVKDSECITKLYHSAVDMTRDIASGLIGPLLVCKHKALSVKGVQNKADVEQHAVFAVFDENKSWYLEDNIKKYCSNPSAVKKDDPK
Pseutarin C     LDTDEPTVKDSECITKLYHSAVDMTRDIASGLIGPLLVCKHKALSVKGVQNKADVEQHAVFAVFDENKSWYLEDNIKKYCSNPSAVKKDDPK
Omicarin C      LDTDEPTVKDSECITKLYHSAVDMTRDIASGLIGPLLVCKLKALSVKGVQNKADVEQHAVFAVFDENKSWYLEDNIKKYCSNPSSVKKDDPK
Oscutarin C     LDTDEPTVKDSECITKLYHSAVDMTRDIASGLIGPPLVCKRKALSIRGVQNKADVEQHAVFAVFDENKSWYLEDNIKKYCSNPSSVKKDDPK

FV              FYKSNVMYTLNGYASDRTEVLGFHQSEVVEWHLTSVGTVDEIVPVHLSGHTFLSKGKHQDILNLFPMSGESATVTMDNLGTWLLSSWGSCEM
vFV             FYKSNVMYTLNGYASDRTEVLRFHQSEVVQWHLTSVGTVDEIVPVHLSGHTFLSKGKHQDILNLFPMSGESATVTMDNLGTWLLSSWGSCEM
Pseutarin C     FYKSNVMYTLNGYASDRTEVLRFHQSEVVQWHLTSVGTVDEIVPVHLSGHTFLSKGKHQDILNLFPMSGESATVTMDNLGTWLLSSWGSCEM
Omicarin C      FYKSNVMYTLNGYASDRTEVLGFHQSEVVQWHLTSVGTVDEIVPVHLSGHTFLSKGKHQDILNLFPMSGESATVTMDNLGTWLLSSWGSCEM
Oscutarin C     FYKSNVMYTLNGYASDRTEVWGFHQSEVVEWHLTSVGTVDEIVPVHLSGHTFLSKGKHQDILNLFPMSGESATVTMDNLGTWLLSSWGSCEM

FV              SNGMRLRFLDANYDDEDEGNEEEEEDDGDIFADIFIPPEVVKKKEKDPVNFVSDPESDKIAKELGLLDDEDNQE-ESHNVQTEDDEEQLMIA
vFV             SNGMRLRFLDANYDDEDEGNEEEEEDDGDIFADIFIPSEVVKKKEEVPVNFVPDPESDALAKELGLIDDEGNPIIQPRREQTEDDEEQLMKA
Pseutarin C     SNGMRLRFLDANYDDEDEGNEEEEEDDGDIFADIFIPSEVVKKKEEVPVNFVPDPESDALAKELGLIDDEGNPIIQPRREQTEDDEEQLMKA
Omicarin C      SNGMRLRFLDANYDDEDEGNEEEEEDDGDIFADIFSPPEVVKKKEEVPVNFVPDPESDALAKELGLLDDEDNPE-QSRSEQTEDDEEQLMIA
Oscutarin C     SNGMRLRFLDANYDDEDEGNEEEEEDDGDIFADIFNPPEVVIKKEEVPVNFVPDPESDALAKELGLFDDEDNPK-QSRSEQTEDDEEQLMIA

FV              TMLGFRSFKGSVAEEELNLTALALEEDAHASDPRIDSNSARNPDDIAGRYLRTINRGNKRRYYIAAEEVLWDYSPIGKSQVRSRAAKTTFKK
vFV             SMLGLRSFKGSVAEEELKHTALALEEDAHASDPRIDSNSARNPDDIAGRYLRTINRGNKRRYYIAAEEVLWDYSPIGKSQVRSRAAKTTFKK
Pseutarin C     SMLGLRSFKGSVAEEELKHTALALEEDAHASDPRIDSNSARNPDDIAGRYLRTINRGNKRRYYIAAEEVLWDYSPIGKSQVRSRAAKTTFKK
Omicarin C      SVLGLRSFKGSVAEEELKHTALALEEDAHASDPRIDSNSARNSDDIAGRYLRTINRRNKRRYYIAAEEVLWDYSPIGKSQVRSLPAKTTFKK
Oscutarin C     SMLGLRSFKGSVAEEELKHTALALEEDAHASDPRIDSNSAHNSDDIAGRYLRTINRRNKRRYYIAAEEVLWDYSPIGKSQVRSLPAKTTFKK

FV              AIFRSYLDDTFQTPSTGGEYEKHLGILGPIIRAEVDDVIEVQFRNLASRPYSLHAHGLLYEKSSEGRSYDDKSPELFKKDDAIMPNGTYTYV
vFV             AIFRSYLDDTFQTPSTGGEYEKHLGILGPIIRAEVDDVIEIQFRNLASRPYSLHAHGLLYEKSSEGRSYDDKSPELFKKDDAIMPNGTYTYV
Pseutarin C     AIFRSYLDDTFQTPSTGGEYEKHLGILGPIIRAEVDDVIEIQFKNLASRPYSLHAHGLLYEKSSEGRSYDDKSPELFKKDDAIMPNGTYTYV
Omicarin C      AIFRSYLDDTFQTPSTGGEYEKHLGILGPIIRAEVDDVIEVQFRNLASRPYSLHAHGLLYEKSSEGRSYDDNSPELFKKDDAIMPNGTYTYV
Oscutarin C     AIFRSYLDDTFQTPSTGGEYEKHLGILGPIIRAEVDDVIEVQFRNLASRPYSLHAHGLLYEKSSEGRSYDDNSPELFKKDDAIMPNGTYTYV

FV              WQVPPRSGPTDNTEKCKSWAYYSGVNPEKDIHSGLIGPILICQKGMIDKYNRTIDIREFVLFFMVFDEEKSWYFPKSDKSTRAEKLIGVQS-
vFV             WQVPPRSGPTDNTEKCKSWAYYSGVNPEKDIHSGLIGPILICQKGMIDKYNRTIDIREFVLFFMVFDEEKSWYFPKSDKSTCEEKLIGVQS-
Pseutarin C     WQVPPRSGPTDNTEKCKSWAYYSGVNPEKDIHSGLIGPILICQKGMIDKYNRTIDIREFVLFFMVFDEEKSWYFPKSDKSTCEEKLIGVQS-
Omicarin C      WQVPPRSGPTDNTEKCKSWAYYSGVNPEKDIHSGLIGPILICQKGMIDKYNRTIDIREFVLFFMVFDEEKSWYFPKSDKSTCEEKLIGVQSS
Oscutarin C     WQVPPRSGPTDNTEKCKSWAYYSGVNPEKDIHSGLIGPILICQKGMIDKYNRTIDIREFVLFFMVFDEEKSWYFPKSDKSTCEEKLIGVQS-

FV              RHTFPAINGIPYQLQGLTMYKDENVHWHLLNMGGPKDIHVVNFHGQTFTEEGREDNQLGVLPLLPGTFASIKMKPSKIGTWLLETEVGENQE
vFV             LHTFPAINGIPYQLQGLTMYKDENVHWHLLNMGGPKDIHVVNFHGQTFTEEGREDNQLGVLPLLPGTFASIKMKPSKIGTWLLETEVGENQE
Pseutarin C     LHTFPAINGIPYQLQGLTMYKDENVHWHLLNMGGPKDIHVVNFHGQTFTEEGREDNQLGVLPLLPGTFASIKMKPSKIGTWLLETEVGENQE
Omicarin C      HHTFPAINGIPYQLQGLMMYKDENVHWHLLNMGGPKDIHVVNFHGQTFTEEGREDNQLGVLPLLPGTFASIKMKPSKIGTWLLETEVGENQE
Oscutarin C     RHTFPAINGIPYQLQGLMMYKDENVHWHLLNMGGPKDVHVVNFHGQTFTEEGREDNQLGVLPLLPGTFASIKMKPSKIGTWLLETEVGENQE

FV              RGMQALFTVIDKDCKLPMGLASGIIQDSQISASGHVGYWEPKLARLNNTGKYNAWSIIKKEHEHPWIQIDLQRQVVITGIQTQGAMQLLKHL
vFV             RGMQALFTVIDKDCKLPMGLASGIIQDSQISASGHVGYWEPKLARLNNTAIFNAWSIIKKEHEHPWIQIDLQRQVVITGIQTQGTVQLLQHS
Pseutarin C     RGMQALFTVIDKDCKLPMGLASGIIQDSQISASGHVGYWEPKLARLNNTGKYNAWSIIKKEHEHPWIQIDLQRQVVITGIQTQGTVQLLQHS
Omicarin C      RGMQALFTVIDKDCKLPMGLASGIIQDSQISASGHVEYWEPKLARLNNTGMFNAWSIIKKEHEHPWIQIDLQRQVVITGIQTQGTVQLLKHS
Oscutarin C     RGMQALFTVIDKDCKLPMGLASGIIQDSQISASGHVGYWEPKLARLNNTGMFNAWSIIKKEHEHPWIQIDLQRQVVITGIQTQGTVQLLKHS

FV              YTVEYFFTYSKDGQNWITFKGRHSETQMHFEGNSDGTTVKENHIDPPIIARYIRLHPTKFHNRPTFRIELLGCEVEGCSVPLGMESGAIKNS
vFV             YTVEYFVTYSEDGQNWITFKGRHSETQMHFEGNSDGTTVKENHIDPPIIARYIRLHPTKFYNRPTFRIELLGCEVEGCSVPLGMESGAIKNS
Pseutarin C     YTVEYFVTYSEDGQNWITFKGRHSETQMHFEGNSDGTTVKENHIDPPIIARYIRLHPTKFYNRPTFRIELLGCEVEGCSVPLGMESGAIKNS
Omicarin C      YTVEYFVTYSKDGQNWITFKGRHSETQMHFEGNSDGTTVKENHIDPPIIARYIRLHPTKFYNTPTFRIELLGCEVEGCSVPLGMESGAIKNS
Oscutarin C     YTVEYFVTYSKDGQNWITFKGRHSKTQMHFEGNSDGTTVKENHIDPPIIARYIRLHPTKFYNTPTFRIELLGCEVEGCSVPLGMESGAIKDS

FV              EITASSYKKTWWSSWEPSLARLNLKGRTNAWQPKVNNKDQWLQIDLQHLTKITSIITQGATSMTTSMYVKTFSIHYTDDNSTWKPYLDVRTS
vFV             EITASSYKKTWWSSWEPFLARLNLEGGTNAWQPEVNNKDQWLQIDLQHLTKITSIITQGATSMTTSMYVKTFSIHYTDDNSTWKPYLDVRTS
Pseutarin C     EITASSYKKTWWSSWEPSLARLNLEGGTNAWQPEVNNKDQWLQIDLQHLTKITSIITQGATSMTTSMYVKTFSIHYTDDNSTWKPYLDVRTS
Omicarin C      EITASSYKKTWWSSWEPFLARLNLEGGTNAWQPEVNNKDQWLQIDLQHLTKITSIITQGATSMTTAMYVKTFSIHYTDDNSTWKPYLDVRTS
Oscutarin C     EITASSYKKTWWSSWEPFLARLNLKGRTNAWQPKVNNKDQWLQIDLQHLTKITSIITQGATSMTTSMYVKTFSIHYTDDNSTWKPYLDVRTS

FV              MEKVFTGNINSDGHVKHFFKPPILSRFIRIIPKTWNQYIALRIELFGCEVF
vFV             MEKVFTGNINSDGHVKHFFKPPILSRFIRIIPKTWNQYIALRIELFGCEVF
Pseutarin C     MEKVFTGNINSDGHVKHFFKPPILSRFIRIIPKTWNQYIALRIELFGCEVF
Omicarin C      MEKVFTGNINSDGHVKHFFKPPILSRFIRIIPKTWNQYIALRIELFGCEVF
Oscutarin C     MEKVFTGNINSDGHVKHFFNPPILSRFIRIIPKTWNQYIALRIELFGCEVF
```

F Coagulation factor X

```
Omicarin C      NVFLKSKVANRFLQRTKRANSLFEEFRSGNIERECIEERCSKEEAREVFEDDEKTETFWNVYVDGDQCSSNPCHYRGTCKDGIGSYTCTCLF
Oscutarin C     NVFLKSKVANRFLQRTKRANSLYEEFRSGNIERECIEERCSKEEAREVFEDDEKTETFWNVYVDGDQCSSNPCHYRGTCKDGIGSYTCTCLS
FX isoform 2    NVFLKSKVANRFLQRTKRANSLVEEFKSGNIERECIEERCSKEEAREAFEDDEKTETFWNVYVDGDQCSSNPCHYRGICKDGIGSYTCTCLS
```

```
vFX                        NVFLKSKVANRFLQRTKRANSLVEEFKSGNIERECIEERCSKEEAREVFEDDEKTETFWNVYVDGDQCSSNPCHYRGICKDGIGSYTCTCLS
Pseutarin C                NVFLKSKVANRFLQRTKRANSLVEEFKSGNIERECIEERCSKEEAREVFEDDEKTETFWNVYVDGDQCSSNPCHYRGICKDGIGSYTCTCLS
Tr FX                      NVFLKSKVANRFLQRTKRANSLFEEFKAGNIERECIEERCSKEEAREAFEDNEKTETFWNVYVDGDQCSSNPCHYGGTCKDGIGSYTCTCLA
FX isoform 1               NVFLKSKVANRFLQRTKRANSLFEEFKSGNIERECIEERCSKEEAREAFEDDEKTETFWNVYVDGDQCSSNPCHYGGTCKDGIGSYTCTCLA
Porpharin D                NVFLKSKEANRFLQRTKRSNSLFEEFRPGNIERECIEEKCSKEEAREIFKDNEKTEAFWNVYVDGDQCSSNPCHYGGTCKDGIGSYTCTCLP
Notecarin D                NVFLKSKVANRFLQRTKRSNSLFEEIRPGNIERECIEEKCSKEEAREVFEDNEKTETFWNVYVDGDQCSSNPCHYRGTCKDGIGSYTCTCLP
Trocarin D                 NVFLKSKVANRFLQRTKRSNSLFEEIRPGNIERECIEEKCSKEEAREVFEDNEKTETFWNVYVDGDQCSSNPCHYRGTCKDGIGSYTCTCLP

Omicarin C                 GYEGKNCERVLYKSCRVDNGNCWHFCKPVQNDIQCSCAEGYLLGEDGHSCVAGGNFSCGRNIKTRNKREASLPDF-----------------
Oscutarin C                GYEGKNCERVLYKSCRVDNGNCWHFCKPVQNDIQCSCAEGYLLGEDGHSCVAGGNFSCGRNIKTRNKREASLPDF-----------------
FX isoform 2               GYEGKNCERVLYKSCRVDNGNCWHFCKHVQNDIQCSCAEGYLLGEDGHSCVAGGNFSCGRNIKTRNKREANLPDF-----------------
vFX                        GYEGKNCERVLYKSCRVDNGNCWHFCKSVQNDIQCSCAEGYLLGEDGHSCVAGGNFSCGRNIKTRNKREASLPDF-----------------
Pseutarin C                GYEGKNCERVLYKSCRVDNGNCWHFCKSVQNDIQCSCAEGYLLGEDGHSCVAGGNFSCGRNIKTRNKREASLPDF-----------------
Tr FX                      GYEGKNCQYVLYQSCRVDNGNCWHFCKPVQNEIQCSCAESYLLGDDGYSCVAGGDFSCGRNIKARNKREASLPDFQTDFSDDYDAIDENNFV
FX isoform 1               GYEGKNCEYVLYKSCRVDNGDCWHFCKPVQNGIQCSCAESYLLGEDGHSCVAGGDFSCGRNIKTRNKREANLPDFQTDFSDDYDEIDENNFV
Porpharin D                NYEGKNCEHLLFKSCRFFNGNCWHFCKPVQNDTQCSCAESYRLGDDGHSCVAEGDFSCGRNIKARNKREASLPDF-----------------
Notecarin D                NYEGKNCEKVLYQSCRVDNGNCWHFCKRVQSETQCSCAESYRLGVDGHSCVAEGDFSCGRNIKARNKREASLPDF-----------------
Trocarin D                 NYEGKNCEKVLYQSCRVDNGNCWHFCKRVQSETQCSCAESYRLGVDGHSCVAEGDFSCGRNIKARNKREASLPDF-----------------

Omicarin C                 ------------VQSQNATLLKKSDNPSPDIRIVNGMDCKLGECPWQAVLVDEKEGVFCGGTILSPIYVLTAAHCINQTEKISVVVGEIDKS
Oscutarin C                ------------VQSQNAILLKKSDNPSPDIRIVNGMDCKLGECPWQAVLVDEKEDAFCGGTILSPIYVLTAAHCINQTKMISVVVGEINIS
FX isoform 2               ------------VQSQNATLLKKSDNPSPDIRIVNGMDCKLGECPWQAALVDEKEGVFCGGTILSPIYVLTAAHCINETETISVVVGEIDKS
vFX                        ------------VQSQNATLLKKSDNPSPDIRIVNGMDCKLGECPWQATLVDEKEGVFCGGTILSPIYVLTAAHCFNETETISVVVGEIDKS
Pseutarin C                ------------VQSHNATLLKKSDNPSPDIRIVNGMDCKLGECPWQAALVDDKKGVFCGGTILSPIYVLTAAHCINETETISVVVGEIDRS
Tr FX                      ETPTNFSGLVPTVQSQNATLLKKSDNPSPDIRVVNGTDCKLGECPWQALLINDQGDGFCGGTILSPIYVLTAAHCINQTKYIRVVVGEIDIS
FX isoform 1               ETPTNFSGLVLTVQSQNATLLKKSDNPSPDIRVVNGTDCKLGECPWQALLLNDEGDGFCGGTILSPIYVLTAAHCINQTKYITVVVGEIDIS
Porpharin D                ------------VQSQNATLLKKSDNPSPDIRIINGMDCKLGECPWQAVLLDKEGDVFCGGTILSPIYVLTAAHCITQSKHISVVVGEIDIS
Notecarin D                ------------VQSQKATLLKKSDNPSPDIRIVNGMDCKLGECPWQAVLINEKGEVFCGGTILSPIHVLTAAHCINQTKSVSVIVGEIDIS
Trocarin D                 ------------VQSQKATLLKKSDNPSPDIRIVNGMDCKLGECPWQAVLINEKGEVFCGGTILSPIHVLTAAHCINQTKSVSVIVGEIDIS

Omicarin C                 RVETGHLLSVDKIYVHKKFVPPKKGYKFYEKFDLVSYDYDIAIIQMKTPIQFSENVVPACLPTADFANQVLMKQDFGIISGFGRIFEKGPKS
Oscutarin C                RKNPGRLLSVDKIYVHQKFVPPKKGYEFYEKFDLVSYDYDIAILQMKTPIQFSENVVPACLPTADFANQVLMKQDFGIVSGFGRIFEKGPQS
FX isoform 2               RIETGPLLSVDKIYVHKKFVPPQKAY----KFDLAAYDYDIAIIQMKTPIQFSENVVPACLPTADFANQVLMKQDFGIVSGFGRIFEKGPKS
vFX                        RVETGPLLSVDKIYVHKKFVPPKRGYKFYEKFDLVSYDYDIAIIQLKTPIQFSENVVPACLPTADFANQVLMKQDFGIVSGFGHIFERGPKS
Pseutarin C                RAETGPLLSVDKVYVHKKFVPPKKSQEFYEKFDLVSYDYDIAIIQMKTPIQFSENVVPACLPTADFANQVLMKQDFGIVSGFGGIFERGPNS
Tr FX                      RKKTGRLLSVDKIYVHQKFVP-------------STYDYDIALIQMKTPIQFSENVVPACLPTADFANQVLMKQDFGIVSGFGRTRERGQTS
FX isoform 1               SKKTGRLHSVDKIYVHQKFVP-------------ATYDYDIAIIQLKTPIQFSENVVPACLPTADFANQVLMKQNFGIVSGFGRTRERGKTS
Porpharin D                RKETRHLLSVDKAYVHTKFV-------------LATYDYDIAIIQLKTPIQFSENVVPACLPTADFANQVLMKQDFGIISGFGHTRSGGQTS
Notecarin                  RKETRRLLSVDKIYVHTKFVPPNYYY-VHQNFDRVAYDYDIAIIRMKTPIQFSENVVPACLPTADFANEVLMKQDSGIVSGFGRIRFKEPTS
Trocarin D                 RKETRRLLSVDKIYVHTKFVPPNYYY-VHQNFDRVAYDYDIAIIRMKTPIQFSENVVPACLPTADFANEVLMKQDSGIVSGFGRIQFKQPTS

Omicarin C                 NTLKVLKVPYVDRHTCMVSSESPITPTMFCAGYDTLPRDACQGDSGGPHITAYRDTHFITGIVSWGEGCAKKGKYGIYTKVS
Oscutarin C                KTLKVLKVPYVDRHTCMLSSESPITPTMFCAGYDTLPRDACQGDSGGPHITAYRDTHFITGIVSWGEGCAQTGKYGVYTKVS
FX isoform 2               KTLKVLKVPYVDRHTCMVSSETPITPNMFCAGYDTLPRDACQGDSGGPHTTVYRDTHFITGIVSSGEGCARNGKYGIYTKLS
vFX                        KTLKVLKVPYVDRHTCMLSSNFPITQNMFCAGYDTLPQDACQGDSGGPHITAYRDTHFITGIVSWGEGCAQTGKYGVYTKVS
Pseutarin C                KTLKVLKVPYVDRHTCMLSSNFPITPTMFCAGYDTLPQDACQGDSGGPHITAYRDTHFITGIVSWGEGCARKGRYGIYTKLS
Tr FX                      NTLKVVTLPYVDRHTCMLSSNFPITQNMFCAGYNTLPQDACQGDSGGPHITAYRDTHFITGIISWGEGCAQTGKYGAYTKVS
FX isoform 1               NTLKVVTLPYVDRHTCMLSSNFPITQNMFCAGYDTLPQDACQGDSGGPHITAYRDTHFITGIVSWGEGCAQTGKYGVYTKVS
Porpharin D                NTLKVVTIPYVDRHTCMLSSDFRITPNMFCAGYDTLPRDACQGDSGGPHITAYRDTHFITGIISWGEGCAKKGKYGVYTKVS
Notecarin D                NTLKVITVPYVDRHTCMLSSDFRITQNMFCAGYDTLPQDACQGDSGGPHITAYRDTHFITGIISWGEGCARKGKYGVYTKVS
Trocarin D                 NTLKVITVPYVDRHTCMLSSDFRITQNMFCAGYDTLPQDACQGDSGGPHITAYRDTHFITGIISWGEGCARKGKYGVYTKVS

Omicarin C                 KFILWIKRIMRQKLPSTESSTGRL
Oscutarin C                KFILWIKRIMRQKLPSTESSTGRL
FX isoform 2               KFIPWIKRIMRQKLPSTESSTGRL
vFX                        KFIPWIKRIMRQKLPSTESSTGRL
Pseutarin C                KFIPWIKRIMRQKLPSTESSTGRL
Tr FX                      RFILWIKRIMRLKLPSTESSTGRL
FX isoform 1               KFILWIKRIIRQKQPSTESSTGRL
Porpharin D                NFIPWIKAVMRKHQPSTESSTGRL
Notecarin                  RFIPWIKKIMSLK-----------
Trocarin D                 KFIPWIKKIMSLK-----------
```

Supplemental Figure 2. Multiple sequence alignments of all major venom protein sequences translated from *Pseudonaja textilis* venom gland transcripts. A) Alignment of protein sequences from *P. textilis* three-finger toxin transcripts (3FTx_1 - 3FTx_14; this study), Pseudonaja_LC (A8HDK6; long neurotoxin 1), Pseudonajatoxin b (P13495), Pseudonajatoxin b homolog (Q9W7J5), short-chain neurotoxin 1/ 5 (P.textilis_SC1/5; Q9W7K2), short-chain neurotoxin 2 (P.textilis_SC2; Q9W7K1), short-chain neurotoxin 3 (P.textilis_SC3; Q9W7K0), short-chain neurotoxin 4 (P.textilis_SC4; Q9W7J9), short-chain neurotoxin 6 (P.textilis_SC6; Q9W7J7), short-chain neurotoxin 7 (P.textilis_SC7; Q9W7J6), and short-chain neurotoxin 8 (P.textilis_SC8; A8HDK1). Three-finger toxin sequences (long-chain) from Australian species *Austrelaps superbus* (A8S6A8), *Demansia vestigiata* (A6MFK4), *Drysdalia coronoides* (F8J2D7), *Notechis scutatus* (P01384), and *Oxyuranus microlepidotus* (A7X4Q3). Three-finger toxin sequences (short-chain) from Australian species *D. coronoides* (F8J2G3) and *O. microlepidotus* (A7X4S7).

B) Alignment of protein sequences from cysteine-rich secretory proteins from *P. textilis* (CRISP_1; this study), *P. textilis* pseudechetoxin-like (Q3SB05), and Australian species *A. superbus* (A8S6B6), *D. coronoides* (F8J2D4), *N. scutatus* (Q3SB04), *O. microlepidotus* (Q3SB06), *P. australis* pseudechetoxin (Q8AVA4), and *P. porphyriacus* (Q8AVA3). C) Alignment of protein sequences from Kunitz-type serine protease inhibitors from *P. textilis* venom transcripts (KUN_1-4; this study), textilinin-1 (Q90WA1), textilinin-2 (Q90WA0), textilinin-3 (Q90W99), textilinin-4 (Q90W98), textilinin-5 (Q90W97), textilinin-6 (Q90W96), textilinin-7 (B5L5Q1), and Australian species *O. microlepidotus* (Microlepidin-3; B5KL27), *O. scutellatus* (Scutellin-3; B5KL29) and *Pseudechis australis* (Mulgin-3; Q6ITB9). D) Alignment of protein sequences from *P. textilis* phospholipase A_2 (PLA_2) transcripts (PLA2_1-3; this study), textilotoxin subunit A (P23026), textilotoxin subunit B (P23027), textilotoxin subunit C (P30811), textilotoxin subunit D (P23028), *P. textilis* acidic phospholipase A_2 1 (Pseudonaja_1; Q9W7J4), *P. textilis* acidic phospholipase A_2 2 (Pseudonaja_2; Q9W7J3), and PLA_2s from Australian species *A. superbus* (Q9PUG7), *N. scutatus* (Q9PSN5), *Oxyuranus scutellatus* (Q4VRI5), and *Tropidechis carinatus* (Q45Z25). E) Alignment of protein sequences from pseutarin C venom factor V (vFV; this study), characterized pseutarin C venom factor V (Q7SZN0), *P. textilis* coagulation factor V (Q593B6), and non-catalytic subunits of prothrombin activators in *O. microlepidotus* (omicarin C; Q58L90) and *O. scutellatus* (oscutarin C; Q58L91). F) Alignment of protein sequences from pseutarin C venom factor X (vFX; this study), characterized pseutarin C venom factor X (Q56VR3), *P. textilis* coagulation factor X isoform 1 (FX isoform 1; Q1L659), *P. textilis* coagulation factor X isoform 2 (FX isoform 2; Q1L658), *T. carinatus* coagulation factor X (Tr FX; Q4QXT9) and catalytic subunits of prothrombin activators in *N. scutatus* (Notecarin D1; P82807), *O. microlepidotus* (omicarin C; Q58L95), *O. scutellatus* (oscutarin C; Q58L96), *Pseudechis porphyriacus* (porpharin D; Q58L93), and *T. carinatus* (trocarin D; P81428).

Supplemental Figure 3

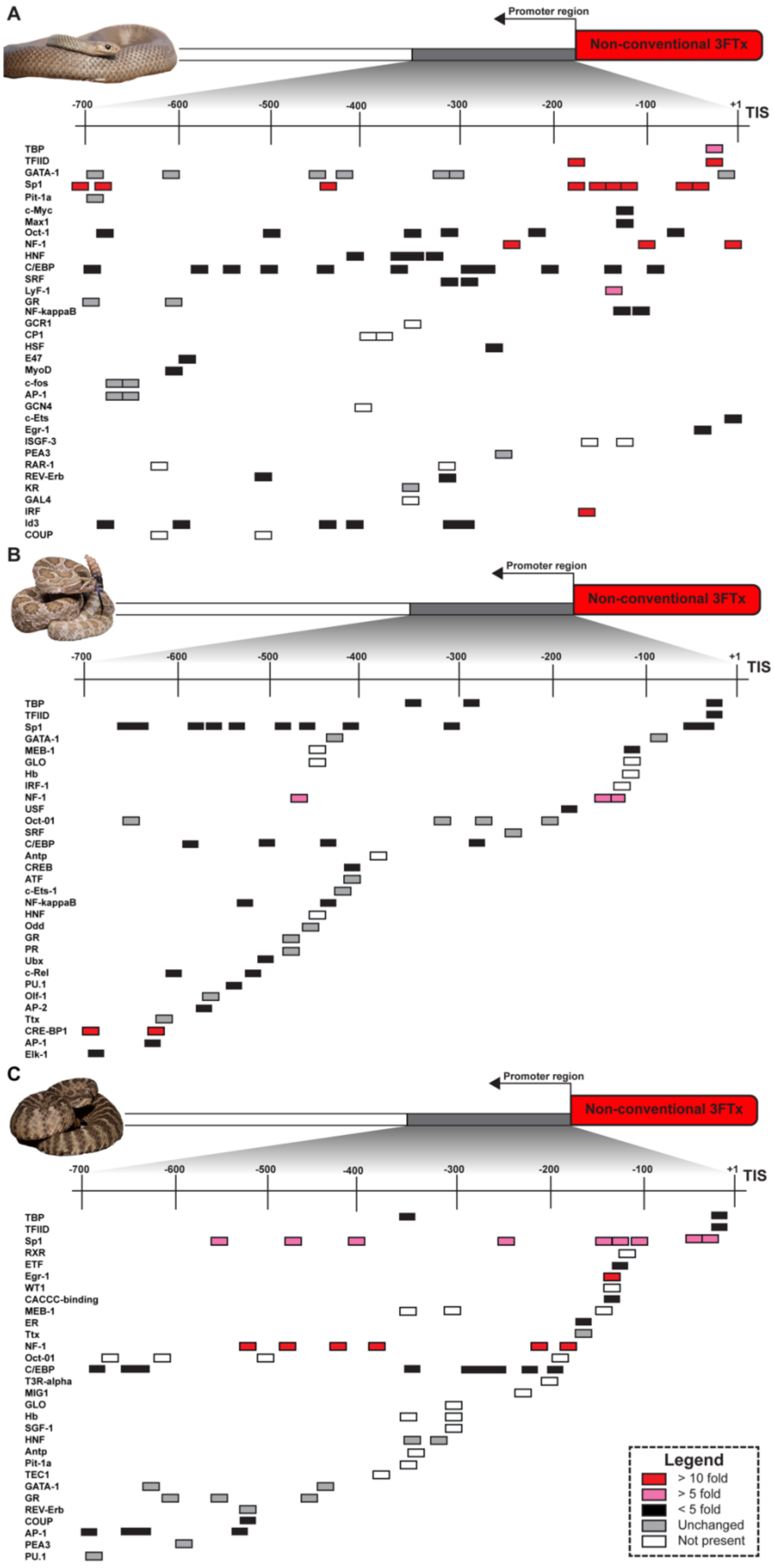

Supplemental Figure 3. Predicted *cis*-regulatory elements in the promoter regions of non-conventional three-finger toxin genes and homologs in an elapid and viperids. *Cis*-regulatory elements (CREs) were predicted 713 bp upstream from what would be the transcription initiation site (TIS) for the (A) *Pseudonaja textilis* non-conventional three-finger toxin (3FTx) (XP_026561523) present in the genome. Non-conventional 3FTxs or plesiotypic 3FTx-like homologs are present in viperid genomes, and 700 bp upstream from what would be the TIS of the closest *P. textilis* non-conventional 3FTx homologs in (B) *Crotalus viridis* and (C) *C. tigris* (XM_039332037.1; LOC120302985) were also evaluated for CREs. Fold-changes in expression levels of *trans*-factors known to interact with predicted CREs are shown between the *P. textilis* and viperid milked and unmilked venom glands (96 hours post venom milking). CRE predictions were completed with the online server AliBaba2.1 using the TRANSFAC 4.0 database. Photo credits: *P. textilis,* Ákos Lumnitzer; *C. viridis,* Wolfgang Wüster; *C. tigris,* Ben Lowe.

Supplemental Figure 4

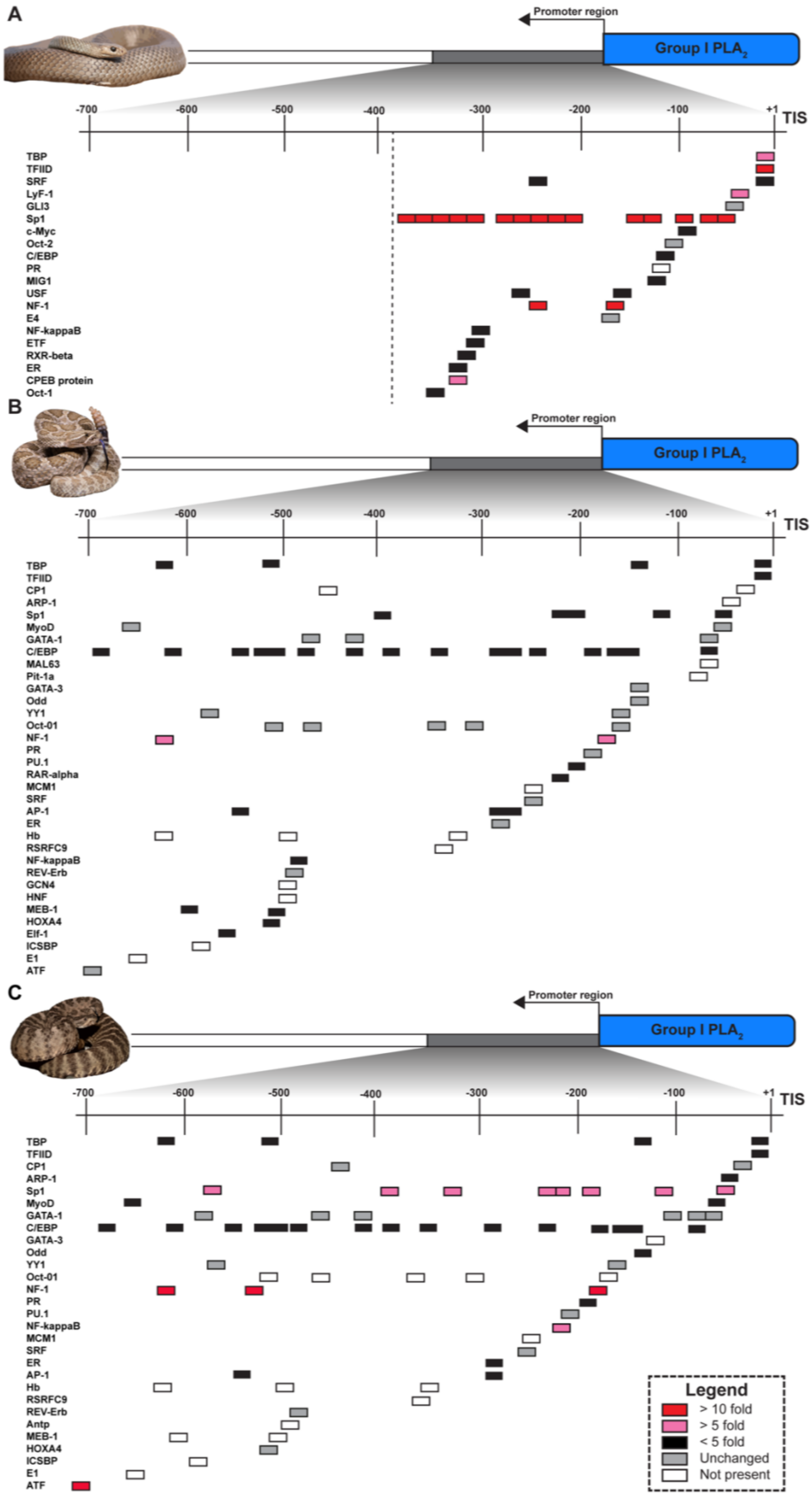

Supplemental Figure 4. Predicted *cis*-regulatory elements in the promoter regions of group I phospholipase A_2 genes and homologs in an elapid and viperids. From the *Pseudonaja textilis* genome, 385 bp upstream from the transcription initiation site (TIS) for (A) group IB phospholipase A_2 (PLA_2) in *P. textilis* (AY027495) was evaluated for *cis*-regulatory elements (CREs). Group I PLA_2 homologs are present in viperid genomes, and 700 bp upstream from what would be the TIS of the closest *P. textilis* group I PLA_2 (AY027495) homologs in (B) *Crotalus viridis* and (C) *C. tigris* (XM_039332037.1; LOC120302985) were also evaluated for CREs. Fold-changes in expression levels of *trans*-factors known to interact with predicted CREs are shown between the *P. textilis* and viperid milked and unmilked venom glands (96 hours post venom milking). CRE predictions were completed with the online server AliBaba2.1 using the TRANSFAC 4.0 database. Photo credits: *P. textilis*, Ákos Lumnitzer; *C. viridis*, Wolfgang Wüster; *C. tigris*, Ben Lowe.

Supplemental Figure 5

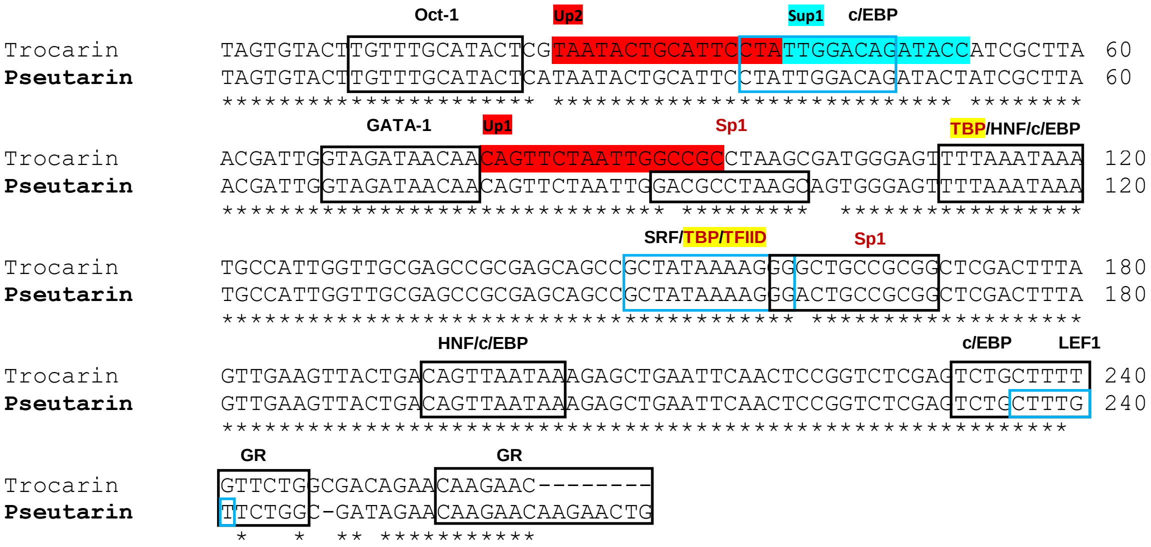

Supplemental Figure 5. Alignments of the two *VERSE* core promoter sequences from pseutarin C (*Pseudonaja textilis*) and trocarin D (*Tropidechis carinatus*) with predicted *cis*-regulatory elements. Three regulatory regions have been identified in this *VERSE* core promoter, two that upregulate venom factor X expression (Up1 and Up2, highlighted in red) and one that suppresses expression (Sup1, highlighted in teal) [40]. *Cis*-regulatory element (CRE) predictions were completed with the online server AliBaba2.1 using the TRANSFAC 4.0 database. *Trans*-regulatory factors that bind to CREs that were upregulated more than 10-fold in the venom gland of *P. textilis* after venom milking are colored in red.

Supplemental Figure 6

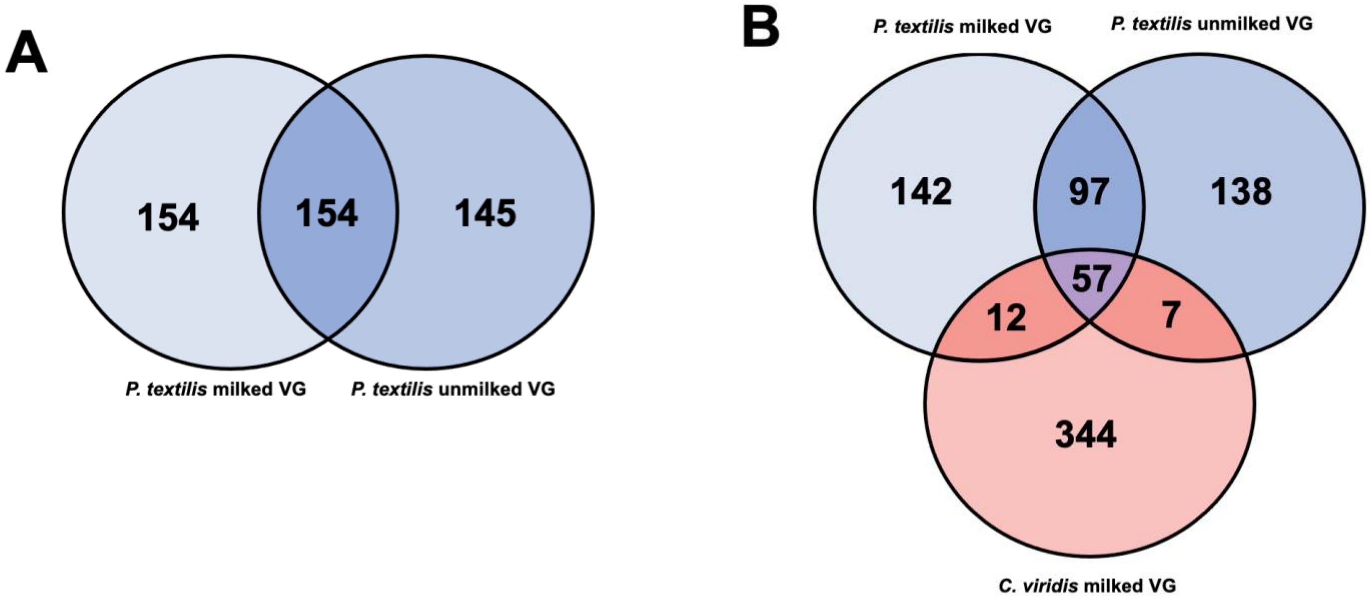

Supplemental Figure 6. Conservation of microRNAs between milked and unmilked *Pseudonaja textilis* venom glands and a milked *Crotalus viridis* venom gland. (A) There were approximately equal numbers of common and unique mature miRNA sequences in the milked and unmilked *P. textilis* venom glands. (B) Only 76 miRNAs were common to both *P. textilis* venom glands and the *Crotalus viridis* venom gland. VG = venom gland.

Supplemental Figure 7

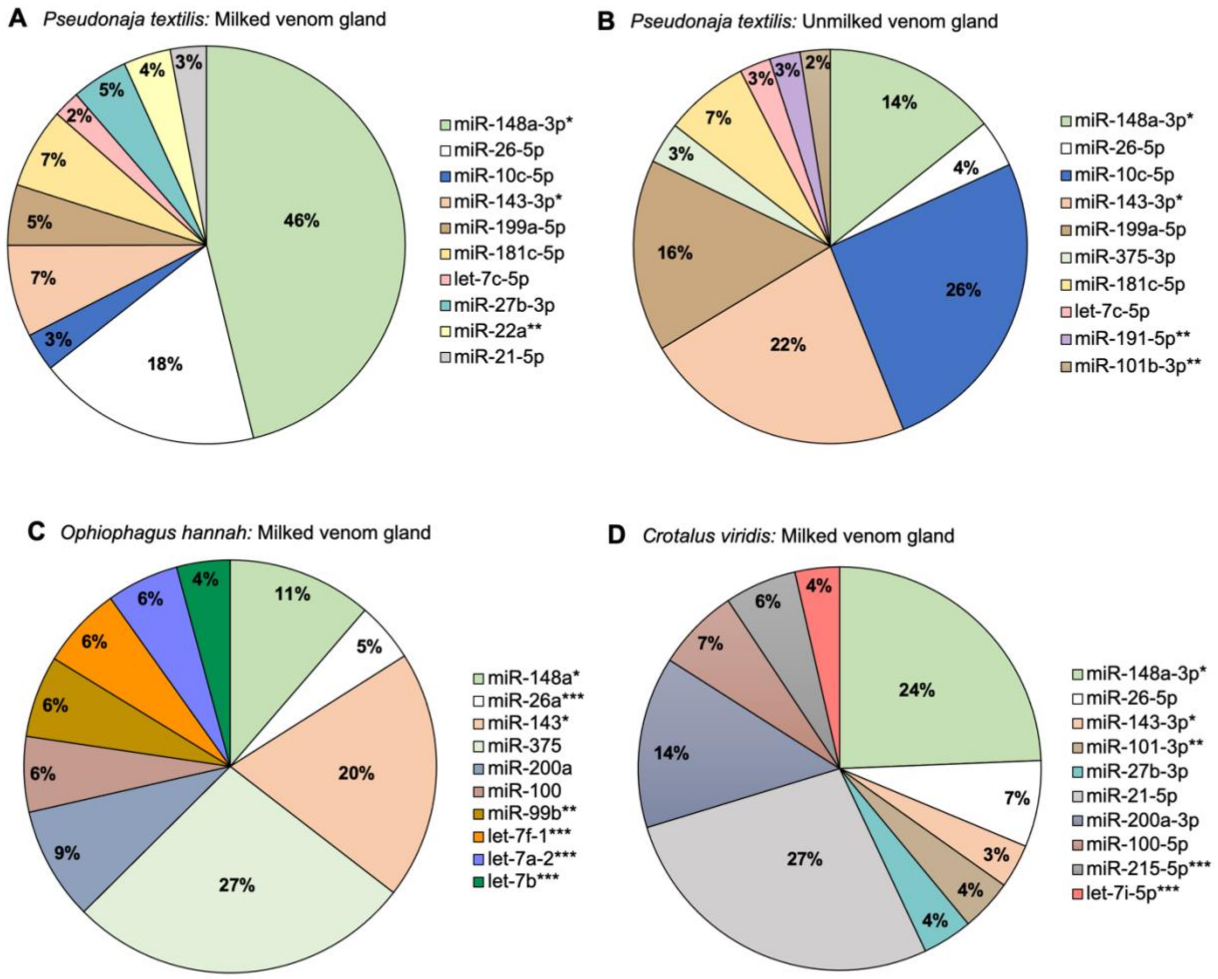

Supplemental Figure 7. Abundances of the top ten expressed miRNAs in snake venom glands. Shown are the expression percentages for each of the top ten miRNAs in the (A) *Pseudonaja textilis* milked venom gland, (B) *P. textilis* unmilked venom gland, (C) *Ophiophagus hannah* milked venom gland, and (D) *Crotalus viridis* milked venom gland. * = miRNAs that are in the top ten most abundant miRNAs for all species, ** = miRNAs shared between species, but not in the top ten for all, and *** = miRNAs that are species-specific.

Supplemental Figure 8

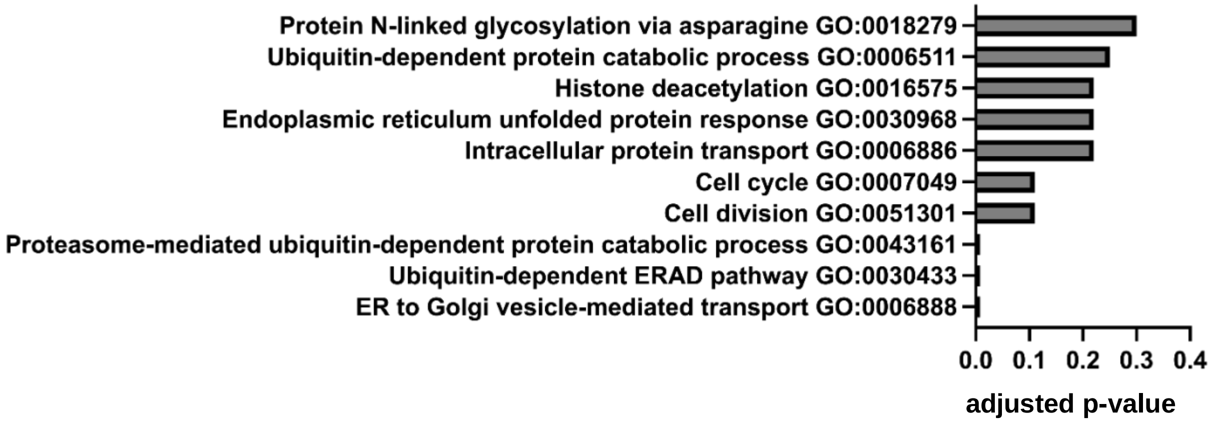

Supplemental Figure 8. Biological processes associated with the transcripts targeted by *Pte-miR-1*. Analysis was completed using DAVID Bioinformatics Resources [73, 74] and Benjamini-Hochberg adjusted p-values used for identifying levels of significance for each biological process.
